# Three Novel Players: PTK2B, SYK, and TNFRSF21 Were Identified to Be Involved in the Regulation of Bovine Mastitis Susceptibility via GWAS and Post-transcriptional Analysis

**DOI:** 10.3389/fimmu.2019.01579

**Published:** 2019-08-06

**Authors:** Fan Yang, Fanghui Chen, Lili Li, Li Yan, Tarig Badri, Chenglong Lv, Daolun Yu, Manling Zhang, Xiaojun Jang, Jie Li, Lu Yuan, Genlin Wang, Honglin Li, Jun Li, Yafei Cai

**Affiliations:** ^1^Anhui Provincial Key Lab of the Conservation and Exploitation of Biological Resources, College of Life Sciences, Anhui Normal University, Wuhu, China; ^2^College of Animal Science and Technology, Nanjing Agricultural University, Nanjing, China; ^3^National Animal Husbandry Station, Beijing, China; ^4^Department of Radiation Oncology, Linyi People Hospital, Linyi, China; ^5^Department of Biochemistry and Molecular Biology, Medical College of Georgia, Augusta University, Augusta, GA, United States

**Keywords:** SNPs, bovine mastitis, protein tyrosine kinase 2 (PTK2B), spleen tyrosine kinase (SYK), TNF receptor superfamily member 21 (TNFRSF21), immune responses

## Abstract

Bovine mastitis is a common inflammatory disease caused by multiple factors in early lactation or dry period. Genome wide association studies (GWAS) can provide a convenient and effective strategy for understanding the biological basis of mastitis and better prevention. 2b-RADseq is a high-throughput sequencing technique that offers a powerful method for genome-wide genetic marker development and genotyping. In this study, single nucleotide polymorphisms (SNPs) of the immune-regulated gene correlative with mastitis were screened and identified by two stage association analysis via GWAS-2b-RADseq in Chinese Holstein cows. We have screened 10,058 high quality SNPs from 7,957,920 tags and calculated their allele frequencies. Twenty-seven significant SNPs were co-labeled in two GWAS analysis models [Bayesian (*P* < 0.001) and Logistic regression (*P* < 0.01)], and only three SNPs (rs75762330, C > T, PIC = 0.2999; rs88640083, A > G, PIC = 0.1676; rs20438858, G > A, PIC = 0.3366) were annotated to immune-regulated genes (PTK2B, SYK, and TNFRSF21). Identified three SNPs are located in non-coding regions with low or moderate genetic polymorphisms. However, independent sample population validation (Case-control study) data showed that three important SNPs (rs75762330, *P* < 0.025, OR > 1; rs88640083, *P* < 0.005, OR > 1; rs20438858, *P* < 0.001, OR < 1) were significantly associated with clinical mastitis trait. Importantly, PTK2B and SYK expression was down-regulated in both peripheral blood leukocytes (PBLs) of clinical mastitis cows and *in vitro* LPS (*E. coli*)–stimulated bovine mammary epithelial cells, while TNFRSF21 was up-regulated. Under the same conditions, expression of Toll-like receptor 4 (TLR4), AKT1, and pro-inflammatory factors (IL-1β and IL-8) were also up-regulated. Interestingly, network analysis indicated that PTK2B and SYK are co-expressed in innate immune signaling pathway of Chinese Holstein. Taken together, these results provided strong evidence for the study of SNPs in bovine mastitis, and revealed the role of SYK, PTK2B, and TNFRSF21 in bovine mastitis susceptibility/tolerance.

## Introduction

Bovine mastitis is the most complex and costly inflammatory disease with high incidence, which seriously affects developing dairy industry worldwide (1–3). Although the breeding conditions of dairy cows have improved, mastitis is still a concern. Dairy cow mastitis causes direct economic losses in many ways, including decreased milk production, increased treatment cost, antibiotic residues, bacterial contamination, and even increased elimination rate due to cow death (4–7). Previous studies have shown that cow mastitis has complex traits (clinical and subclinical mastitis) and is affected by multiple factors, including genetic features, nutrition, and hygiene standard, pathogen infections, and seasonal changes. Among them, pathogen infection (Gram-negative, such as *Escherichia coli*; Gram-positive, such as *Staphylococcus aureus* and *Streptococcus uberis*) is the main cause of mastitis (8–13). Therefore, rapid identification of pathogens is critical to determine the symptoms of infection (14). However, mastitis-related immune response is a complex biological process involving immune cells, mammary epithelial cells, and endothelial cells (8). It is well-known that mastitis causes a dramatic increase in bovine milk somatic cell counts (SCC), mainly neutrophils (8, 15, 16). It is also known that somatic cell score (SCS) is the primary trait for detection of mastitis with high hereditary capacity (17). Thus, screening and identifying susceptible or resistant genes associated with SCC or SCS will provide novel strategies to reduce the incidence of mastitis and improve the quality of dairy cows populations (17–19).

It has been recognized that better understanding of the genetic and biological basis of a complex environment will facilitate genome prediction and the development of appropriate control strategies (20–24). In the past decade, different research strategies, including single nucleotide polymorphism (SNP) in a candidate gene, quantitative trait loci (QTL) and GWAS (12, 25, 26), were successfully used to identify the genes significantly associated with the mastitis traits. GWAS has achieved unprecedented success in identifying gene regions and candidate gene variations that are closely related to clinical phenotypes and disease susceptibility (chromosome and gene level, in term of the association between SNPs and traits) (27–29). In addition, identification of associated gene mutations can help reveal the pathogenesis of disease, provide an entry point for treatment, and analyze common genetic variations to identify multiple risk loci for complex diseases (30, 31). GWAS is widely regarded as a potent method to identify SNPs in dairy cattle mastitis traits (17, 32). GWAS data showed that Bos Taurus autosome 2, 4, 6, 10, 14, 18, and 20 associated with clinical mastitis were significantly correlated with SCS in cows (33–35). Additionally, two clinical mastitis candidate genes [vitamin D-binding protein precursor (GC) and neuropeptide FF receptor 2 (NPFFR2)] were detected using high-density single nucleotide polymorphic array and genomic sequencing (18, 26). Wu et al. detected five mastitis susceptibility genes (NPFFR2, SLC4A4, DCK, LIFR, and EDN3) in Danish Holsteins (36). In 2015, Wang et al. identified another two mastitis susceptibility genes (TRAPPC9 and ARHGAP39) in Chinese Holstein (17). In the same year, Wu et al. confirmed the differential expression of TLR4/NF-κB signaling pathway-related genes (up-regulation genes: TLR4, MyD88, IL-6, and IL-10; down-regulation genes: CD14, TNF-α, MD-2, IL-1β, NF-κB, and IL-12) in the mammary gland of Chinese Holstein mastitis cows by Gene-Chip microarray (37). Genetic variations in immune response, specific pathogen (LY75, DPP4, ITGB6, and NR4A2) and lymphocyte antigen-6 complex genes (LY6K, LY6D, LYNX1, LYPD2, SLURP1, and PSCA) might lead to clinical mastitis in American Holstein cows (38). Additionally, single gene polymorphisms (CXCR1, MAP4K4) and their signaling pathways (TLR4/NF-κB) served as genetic markers for mastitis in different cow populations (12, 39). However, there is some inconsistency in genetic variation or polymorphism in genes associated with mastitis traits. Therefore, genetic mutations and polymorphisms in mastitis-associated genes should be screened and validated in different populations.

Type IIB restriction site-associated DNA sequencing (2b-RADseq) is a high-throughput genotyping-by-sequencing (GBS) method based on type IIB restriction endonuclease (for instance, BsaXI and AlfI), which provides a powerful method for identifying gene SNP in the population genome (40, 41). It has strong technical repeatability, uniform depth of sequencing, high cost-effectiveness and genome wide coverage (40, 42). Furthermore, 2b-RADseq technique successfully predicted multi-locus sequence typing (MLST) and provided more details on the population information than MLST technique (41). In addition, this method is also suitable for creating high-density genetic or linkage maps of genomic region or locus markers and revealing the regions associated with related traits by QTL mapping and association analysis (43, 44). More importantly, 2b-RAD can identify key SNPs associated with traits by deep sequencing in fewer samples, thereby preliminary labeling candidate genes (45). Therefore, 2b-RAD may be an ideal genomic screening and labeling platform for detecting mastitis resistance or susceptibility genes in dairy cattle.

In this study, GWAS-2b-RADseq was used to screen and identify mastitis susceptibility or resistance SNPs in Chinese Holstein, and to validate the reliability of key significant SNPs in another independent sample population. Then, immune-regulated or inflammatory associated genes were marked based on the results of significant differential SNPs Gene Ontology (GO) annotation. Finally, the expression levels of candidate genes were evaluated both *in vitro* (peripheral blood leukocytes, PBLs) and *in vivo* (bovine mammary epithelial cells, bMECs). Understanding the genetic and biological pathways of bovine mastitis susceptibility or resistance candidate genes is a worthwhile strategy for anti-mastitis research and application.

## Materials and Methods

### Sample Libraries and Preparation

The procedures involving animals were approved by the Animal Welfare Committee of Nanjing Agricultural University, Nanjing, China, and approval No.20160615. And all animal experiments were carried out in strict accordance with the guidelines and rules established by the committee.

Chinese Holstein cows used for 2b-RADseq library and independent sample population validation came from two different pastures of the Nanjing Weigang Dairy Co., Ltd. At 2b-RADseq stage, forty dairy cows, daughters of ten bulls, were selected from 596 lactating Chinese Holstein in the third early-lactation period (15–60 d), which were further divided into two subgroups according to their clinical mastitis phenotypes [mammary gland (visual observation): red, swollen, fever, pain, and milk flocculation, etc., and laboratory diagnosis (mainly SCC)] (46, 47): case group [20 cows, clinical mastitis appeared in all three lactation periods, and SCC values [(2100.3 ± 891.1) × 10^4^) > 100 × 10^4^ cells/ml] and control group [20 cows, no mastitis during the three lactation periods, and SCC values [(22.4 ± 6.8) × 10^4^) < 30 × 10^4^ cells/ml]. At the independent sample population validation stage, 383 cows (73 in case group and 310 in control group), daughters of thirty-one bulls, were selected from 886 lactating cows in the third early-lactation period (15–60 d) in another pasture for population genetics verification. All experimental cows' mastitis clinical traits were diagnosed by pasture veterinarians, and SCC were obtained from pasture Dairy Herd Improvement (DHI). In their respective pastures, all animals have the same growth, feeding environment, and similar production levels (305 days correction of milk yield, 305D).

Blood samples were aseptically collected from the bovine coccygeal vein to a disposable (EDTA-K2) vacuum anticoagulation tube. And blood samples were collected from the case group during the presence of clinical mastitis based on SCC and clinical findings. Genomic DNA was extracted from whole blood using *TIANamp Genomic DNA Kit* (Cat#DP304-03, TIANGEN Biotech). The quality of genomic DNA was detected by *NanoDrop* and *agarose gel* methods (extracted 3 μL of genomic DNA, loaded on 1% agarose gel, 100 V CV 25 min, viewed under ultraviolet light and photographed). Genomic DNA with a quality (OD_260_/OD_280_ = 1.7–1.8) that meets the experimental requirements was used for subsequent 2b-RADseq analysis.

### 2b-RAD Library, Sequencing, and Raw Reads Quality Control

Forty sample libraries set up met a protocol developed by 2b-RAD sequencing needs with a little change and five-label tandem technique (40, 48). The *Bos Taurus* genomes (*Bos taurus UMD 3.1.1*) was used as the reference for predicting electronic-enzyme-cut digestion of genomic DNA. Finally, *Bael* restriction endonuclease, a commonly used restriction endonuclease that specifically recognize the AC(N)_4_GTAYC (NNNNNNNNNNACNNNNGTAYCNNNNNNNNNNNN) and GRTAC(N)_4_GT (NNNNNNNNNNNNNNNTGNNNNCATRGNNNNNNN) sites, and has the advantages of high stability, good repeatability, and uniform distribution in the genome, was selected to digest genomic DNA. The restriction enzyme digested DNA fragment tags of each sample were linked by standard 5′-NNN-3′ connector. Paired-end sequencing was carried out on the *Illumina Hiseq Xten* (https://support.illumina.com/downloads/sequencing-analysis-viewer-software-v2-1-8.html) platform. Construction the library and steps of the modeling followed Wang et al. with a minor change (41); Supplemental statistical model (Figure S1).

### SNPs RAD Typing, Linkage Disequilibrium (LD) and Genetic Diversity Analysis

Single nucleotide polymorphism marker typing (RAD typing) was performed on Enzyme Reads using the maximum likelihood (ML) method in SOAP software. The statistic SNP typing results, using R Package cluster analysis of the differences between sample SNPs. SNPs were annotated using SnpEff software (Version: 4.1g) (http://snpeff.sourceforge.net/). Plink software was used to calculate the r^2^ value of the pairwise SNPs, with the main parameters set to: -r^2^-Id-window-kb 1000-Id-window 50-Id-window-r^2^ 0.2. According to the median of the software, the work F_(x)_ = 1/(log_10_ ((x+10^(7−C)^)/10^7^) + C) was used to fit, and map by chromosome/grouping. To find the LD block in the case-control group, we added the parameters “block output GAB-pair wise Tagging' to R package runner.

Principal component analysis (PCA) method was used to evaluate the population. Then a correlation test for each subgroup, including the first five PC selected as covariate analysis signs for population division adjustment (Table S2). And the first two influential feature vectors were selected to draw the correlation between the samples. To assess experimental samples' genetic diversity, polymorphic information content (PIC), observed heterozygosity (Ho) and expected heterozygosity (He) values were also calculated for each SNP loci. In addition, the genetic differentiation coefficient between the subgroups was statistical.

### Association Analysis Between SNPs and Clinical Mastitis Traits in Chinese Holstein

We considered a GWAS for the quality traits of dairy cow mastitis, and 2b-RAD markers genotype for each individual SNP loci. To ensure the accuracy of the analysis, we used multi-stage GWAS for identification of important SNPs associated with mastitis traits. GWAS analysis was performed using Bayesian and Logistic regression analysis model to compare significant SNPs between case-control. Quantile-Quantile Plot (QQ-plot) evaluated the rationality of the two analysis models. GO enrichment analysis performed on all genes with SNPs, and their functions described in conjunction with GO annotations. Hypergeometric Distribution Test (*Cytoscape* software 3.6) used to calculate significant gene enrichment in each GO entry. Protein and protein interaction networks were predicted and constructed in the string 10.5 database (Bos Taurus) (49).

### Independent Sample Population Validated Significant SNPs

The independent sample population validation had the same screening conditions as the 2b-RADseq stage. The case-control study was used for the population validation of significant SNPs. The genomic DNA from peripheral blood of cows was extracted by Rapid Blood Genomic DNA Isolation Kit (#B518223, Sangon Biotech). The whole blood genomic DNA quantified (OD260/OD280 = 1.7–1.8) by the SparkTM multimode microplate reader (Tecan, Swizerland). Table S7 lists the PCR primer sequence details containing significant SNPs. The volume of the final PCR reaction system was 50 μl, including 5 μl 10 × Buffer (Mg2+), 1 μl dNTPs, 1.4 upstream, and 1.2 μl downstream primer, 0.4 μl Taq-DNA polymerase, 200 ng DNA template, and 35 μl ddH_2_O. PCR reaction conditions were as follows: denaturation 95°C for 5 min, 32 cycles of 95°C for 40 s, 58°C for 30 s, and 72°C for 50 s; and extension at 72°C for 7 min. PCR products detected by 2% agarose gel electrophoresis, then used for direct sequencing after passing the quality test.

### Blood Total RNA Extraction and Transcriptional Analysis

Total RNA extracted from peripheral of cow blood samples and bMECs using the Whole Blood Total RNA Isolation Kit (#B518623, Sangon Biotech). The concentration of RNA was quantified (OD260/OD280 = 1.9–2.0, 2 μg/μl) by the SparkTM multimode microplate reader. The gDNA removal reaction system was 10 μl, including gDNA Buffer 2 μl, total RNA 4 μl, and RNase-free water 4 μl, 42°C for 5 min. The first strand cDNA extracted from total RNA (4 μl, ~3 μg) using RevertAid First Strand cDNA Synthesis Kit (#K1621, Thermo Scientific). RT-qPCR reaction performed on the Bio-RadCFX ManagerTM (United States) using the SuperReal (SYBR Green) fluorescence quantification kit (#FP205, TIANGEN Biotech). The primer sequence details for RT-qPCR shown in Table S8. The protocol as followed: 95.0°C for 15.0 min; 95.0°C for 20 s, 57.0°C for 20 s, 72.0°C for 30 s, 40 cycles; Melt Curve 65.0°C for 5 s, 95.0°C increment 50 s; 4.0°C forever.

### Isolation and Primary Culture of Bovine Mammary Epithelial Cells (bMECs) From Control Group

The steps of isolation of bMECs were as follows (50, 51): (1) Three healthy Chinese Holstein cows in the third early-lactation stage were selected and slaughtered. Then breast tissues (including mammary acinar tissue) were collected and cleaned with 75% ethanol for two times (5 min/times), and placed in Dulbecco's Modified Eagle's Medium/Nutrient Mixture F-12 Ham (DMEM/F12) complete medium supplemented with 15% Fetal Bovine Serum (FBS, Lot#42F1376K, Gibco, USA), 1% Penicillin-Streptomycin Solution (Double-antibiotics, Cat#C0222, Beyotime, China), 1% Hydrocortisone (Cat#CS-2226, MedChemExpress, USA), and 1% Estradiol (Cat#HY-B0141, MedChemExpress, USA). The breast tissues were brought back to the aseptic processing room within 2 h. (2) Rinse the breast tissue with 1 × PBS buffer (containing double-antibiotics) under sterile conditions until clarified. (3) The mammary acinar tissues were isolated under aseptic conditions, and washed with DMEM/F12 medium until clarification, then centrifuged at 800 rpm for 2 min. (4) Mammary acinar tissues were cut into 1–2 mm^3^ tissue pieces with sterile scissors, rinsed to clear with DMEM/F12 medium, and centrifuged at 800 rpm for 2 min. (5) The tissue pieces were uniformly inoculated into a cell culture flask, placed in a 37°C, 5% CO_2_ incubator, inverted for 30–60 min, and the culture solution was removed. (6) The cell culture flask was placed upright and DMEM/F12 complete medium was added. The culture medium was changed every 48 h, and the bMECs were purified when the cell adhering density reached 90%. (7) Primary culture of bMECs: The purified cell suspension was inoculated into new culture flask, placed in 37°C, 5% CO_2_ cell incubator, and the culture medium was changed every 48 h. (8) Identification of bMECs: Epithelial cells are sensitive to Cytokeratin-18 (C-04, Cat#sc-51582, SANTA CRUZ, USA), and immunofluorescence staining is used to identify bMECs (Figure S7B). bMECs were sub-cultured (primary cells within 20 generations).

### Lipopolysaccharides (LPS, Cat#D8437, Sigma-Aldrich, USA) From *E. coli* Stimulates bMECs for *in vitro* Validation

The post-transcriptional analysis validation experiment was performed by qRT-PCR using β-actin as an internal reference. Total RNA was extracted by All-In-One DNA/RNA/protein Mini-Preps Kit (#BS88003, Sangon Biotech, China) and stored at −80 °C. Three micrograms of total RNA was used for qRT-PCR using the same protocol. bMECs (5 × 10^5^) were sub-cultured in 6-well-plates for 24 h and then stimulated with LPS. First, bMECs were stimulated with different LPS concentrations (100, 50, 25, 12.5, and 0 μg/ml) for 30 min. Then, specific LPS concentration (25 μg/ml) was selected, based on the SYK expression level, and used in time course (control (0 min), 15, 30, 45, 60, and 120 min) to stimulate bMECs (5 × 10^5^).

### Statistical Model Analysis

Linear models are commonly methods for analyzing phenotypic and genotype correlations. Strict quality control is used to remove SNP sites that are not performing well in RAD typing. In this study, Bayesian and Logistic regression models were used to detect significant SNP loci in clinical mastitis traits in dairy cows (Supplemental statistical models). Case-control study validated the population genetics of important SNP loci associated with mastitis traits in dairy cows. Differential expressions of genes relative quantitative Analysis (2^−ΔΔ*Ct*^) are based on qRT-PCR results via Student's *t*-test and one-way ANOVA (and non-parametric).

## Results

### High Quality SNPs Acquisition, RAD Typing and Population Genetic Differentiation

**Figure 1:**
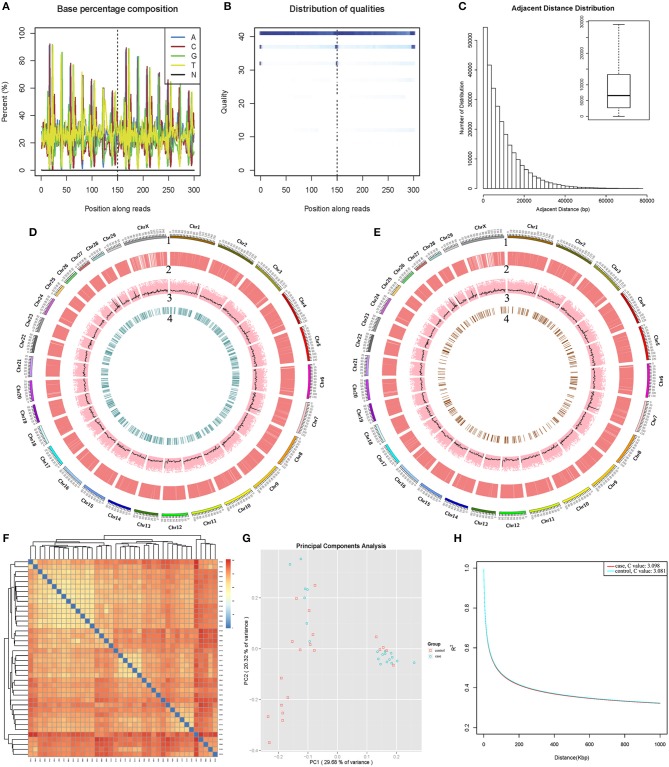
2b-RADseq and biological information analysis of Chinese Holstein cows. **(A)** Proportion of A/C/G/T/N bases at each position: the abscissa was Reads base position, and ordinate were the bases' proportion; different colors represent base types, and the base that was not recognized in sequencing were labeled as N. **(B)** Sequencing base mass value at each position: the darker the color, the higher the base ratio of the mass value in the data. **(C)** Average spacing between adjacent tags: The horizontal line in the middle of the box plot is the average spacing between the labels. **(D)** Bayesian and **(E)** Logistic regression model analysis of SNPs quality traits in all samples (1–4): chromosome scale; SNPs for all samples; sequencing depth for each SNPs; significant SNPs (*P* < 0.05). **(F)** Differential SNP cluster analysis: The color difference indicates the number of difference SNPs between samples and the near-far relationship of clusters between samples. **(G)** Principal Component Analysis and RAD typing of SNPs: Abscissa represented principal component 1 (PC1); ordinate represented principal component 2 (PC2); each point was a sample with different shapes and colors representing different groups. **(H)** The Linkage Disequilibrium attenuation curve of case-control SNPs.

In this project, the genomic DNAs of 40 Chinese Holstein cows digested with *Bael* restriction endonuclease and the total of 7,957,920 unique tags were obtained. On Clean Reads, the proportion of A/C/G/T/N bases appeared in each position were shown in Figures 1A,B was quality values of the base sequencing results at each position and the base recognition accuracy rate were 99.99%. The average distance between adjacent tags was 9,589 bp (Figure 1C), and the unique tag alignment ratio for all samples was 59.69%−72.71%. 10,058 high quality SNPs from 7,957,920 unique tags were selected for RAD typing, and the distribution of SNPs on chromosomes was analyzed by sliding window (Figure S2). All SNPs and their average sequencing depth (17.43 × ) were shown in Figure 1D (Bayesian analysis) and Figure 1E (logistic regression analysis). Differential SNP cluster analysis showed that data pattern similarity within group was higher; while the similarity between groups was lower. The genetic relationship between the samples was also shown in Figure 1F. PCA was performed on the obtained SNP markers to obtain the two most influential feature vectors (Figure 1G).

The LD coefficients between pairwise markers of SNPs in the genome were calculated, and average LD coefficients between the molecular markers of the case and control group were shown in Figure 1H, respectively. Their LD decay rates also appeared to be the same, and *C* value were 3.098 (case) and 3.081 (control). Here, since heterozygote risk assessment was between two homozygotes, this line fit the data reasonably which matched to additive genotype risk. In this case there was no deviation, and the test was convincing (Figure S3A, Table S3). We also calculated the genetic differentiation coefficient (Wright's fixation index, Fst) between the two groups. The value was 0.01869, indicating that the genetic differentiation between case-control groups was smaller.

### Genome Wide Association Analysis

The Quantile-Quantile Plot (QQ-plot) data showed that the *P*-value was consistent with expected values at all SNP site, indicated that the two analysis models matched well (Figures S3B,C). Bayesian analysis has identified 42 significant SNPs with *P* < 0.001 (Table S4), while logistic regression analysis model has selected 51 SNPs with *P* < 0.01 (Table S5). As expected, significant SNPs screened by two analytical models would vary. Total of 27 significant SNPs appeared simultaneously in both analytical models, except these loci with odds ratio (OR) and 95% Confidence Interval (CI) value were not “Na,” and Upper bounds of 95% confidence interval (U95) value were not “Nan” (Table 1). We also noticed that the OR values of the eight SNPs (rs114843903, rs5881560, rs17514753, rs17518215, rs22015301, rs9704351, rs20438858, and rs5088452) were < 1, indicating that these SNPs are associated with mastitis susceptibility traits. However, the OR values of other 19 SNPs, including rs75762330 and rs88640083, were >1, indicating that these SNPs were associated with mastitis resistance.

**Table 1:** Twenty-seven significant SNPs jointly screened by the Bayesian Model and Logical regression analysis model.

**Ref_ID**	**Chromosome**	**Bayesian analysis**					**Logical regression analysis**				
		P^*^	**CHISQ**	**OR**	**L95**	**U95**	P^**^	**OR**	**L95**	**U95**	**STAT**
rs47045687	AC_000159.1	0.000106	15.02	14.21	2.937	68.76	0.004718	11.36	2.106	61.32	2.826
rs114843903	AC_000159.1	0.000628	11.69	0.1678	0.05817	0.4842	0.006341	0.215	0.07128	0.6483	−2.73
rs90835937	AC_000160.1	0.00067	11.57	5.808	2.038	16.55	0.007839	3.838	1.424	10.34	2.659
rs5881560	AC_000162.1	0.000877	11.07	0.1406	0.04076	0.4852	0.005781	0.1412	0.03515	0.5669	−2.76
rs8678060	AC_000162.1	0.000465	12.25	6.929	2.196	21.86	0.00522	5.866	1.695	20.3	2.793
rs37588412	AC_000162.1	0.000842	11.15	6	1.994	18.06	0.009236	4.128	1.42	12	2.603
rs75762330	AC_000165.1	0.000259	9.078	7.07	1.78	28.08	0.00127	6.605	1.496	29.15	2.492
rs88640083	AC_000165.1	0.000192	5.477	9.25	1.047	81.7	0.00314	12	1.248	115.4	2.152
rs17176625	AC_000166.1	0.000512	12.07	6.662	2.185	20.31	0.0069	4.749	1.534	14.71	2.702
rs17514753	AC_000166.1	0.000126	14.7	0.1146	0.03528	0.3725	0.00321	0.03618	0.003978	0.329	−2.947
rs17518215	AC_000166.1	0.000561	11.9	0.1605	0.05443	0.4733	0.005858	0.05012	0.005962	0.4213	−2.756
rs22015301	AC_000166.1	0.000247	13.44	0.1235	0.03669	0.4155	0.002015	0.1079	0.02628	0.4434	−3.088
rs98519900	AC_000166.1	0.000538	11.98	6.25	2.131	18.33	0.007809	3.866	1.428	10.47	2.66
rs14802054	AC_000169.1	0.000684	11.53	6	2.058	17.5	0.005923	8.018	1.821	35.32	2.752
rs25949166	AC_000170.1	2.68E-05	17.64	12.33	3.262	46.63	0.001027	12.78	2.792	58.52	3.283
rs33866959	AC_000171.1	0.000366	12.7	7.222	2.309	22.59	0.00748	4.233	1.47	12.19	2.675
rs48577224	AC_000173.1	0.0003884	12.59	6	2.154	16.71	0.004518	4.881	1.634	14.58	2.84
rs49099498	AC_000173.1	7.08E-05	15.79	14.57	3.059	69.4	0.00866	7.677	1.676	35.16	2.625
rs27017918	AC_000175.1	0.000265	13.31	20.28	2.496	164.8	0.00583	21.89	2.441	196.4	2.757
rs32265465	AC_000175.1	0.000341	12.83	8	2.344	27.31	0.006382	5.728	1.634	20.08	2.727
rs9704351	AC_000177.1	0.000628	11.69	0.09167	0.01908	0.4404	0.001736	0.05793	0.009745	0.3444	−3.132
rs20438858	AC_000180.1	0.00062	11.71	0.1515	0.04821	0.4762	0.009494	0.2394	0.08128	0.7052	−2.594
rs22490040	AC_000180.1	0.000185	13.97	8.061	2.545	25.53	0.00419	6.859	1.836	25.62	2.863
rs28580132	AC_000180.1	0.0005616	11.9	7.143	2.199	23.21	0.008612	4.642	1.477	14.59	2.627
rs3233588	AC_000181.1	0.0006203	11.71	6.6	2.1	20.74	0.001532	12.28	2.603	57.92	3.169
rs1988979	AC_000182.1	8.24E-05	15.5	11.62	2.975	45.43	0.006471	6.124	1.662	22.57	2.723
rs50888452	AC_000187.1	0.000937	10.95	0.1667	0.05543	0.5011	0.008504	0.22	0.07121	0.6795	−2.631

* indicated the P-value calculated by Chi-square (< 0.001);

** *is the t-statistic P-value of the logical regression model (< 0.01)*.

*CHISQ is Chi-square under Chi-square test. STAT is the t-statistic coefficient under the logistic regression model*.

*OR: Estimated odds ratio. L95: Lower bound of 95% confidence interval for odds ratio. U95: Upper bound of 95% confidence interval for odds ratio. Nan: meaningless number. Na: missing value or Not available*.

**Figure 2:**
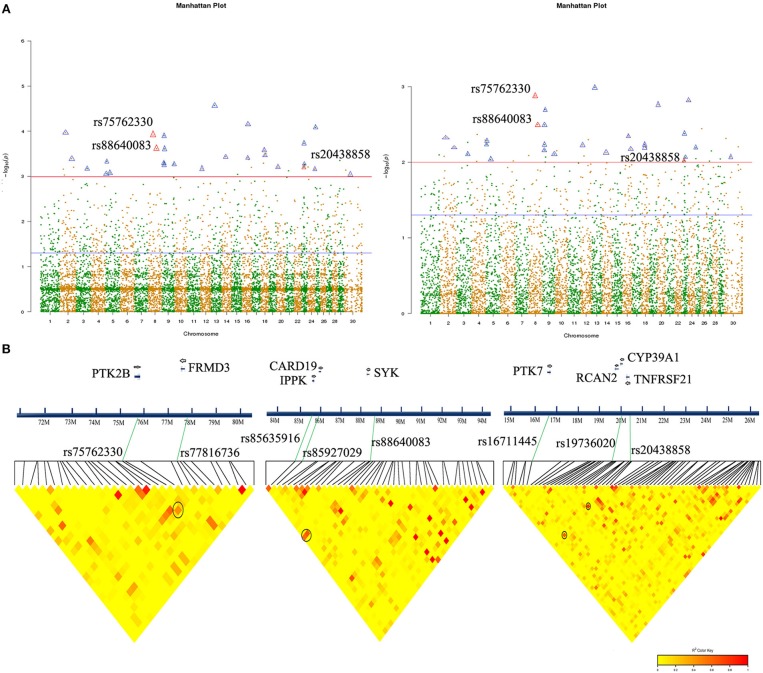
Significant SNP association analysis and chromosomal localization in Chinese Holstein mastitis. **(A)** Manhattan map showed significant SNPs associated with Chinese Holstein mastitis traits (co-marked by both models: Bayesian (left), Logistic analysis (right). Triangular markers are significant SNPs that appear in both analytical models (red triangles are SNPs annotated to immune-related genes: rs75762330, rs88640083, and rs20438858). **(B)** Partial LD block of three significant SNPs, respectively, with a distance interval of 1 Mb. Important SNPs associated with potential candidate functional genes. The redder the LD blocks color, the stronger correlation (black circles).

We annotated all 27 significant SNPs to determine their location in the chromosomal genome (Figure 2A, Table 2):14 SNPs located in the intergenic region, 10 in intron, and one in 3′-UTR, one in upstream and one in downstream. Go annotation for 27 significant SNPs revealed that only three SNPs were annotated to immune-related genes, namely SNPs rs75762330, rs88640083, and rs20438858. SNP rs75762330 (C > T, OR > 1, PIC = 0.2999 > 0.25) was annotated to PTK2B gene located on BTA 8 with moderate polymorphism. SNP rs88640083 (A > G, OR > 1, PIC = 0.1676 < 0.25) annotated to SYK gene located on BTA eight with low polymorphism. SNP rs20438858 (G > A, OR < 1, PIC = 0.3366 > 0.25) was annotated to TNFRSF21 located on BTA 23 and was moderate polymorphism. The Fst values of the rs75762330, rs88640083, and rs20438858 were 0.2165, 0.1297 and 0.2459, respectively (Table S9), suggesting that the genetic differentiation of the three SNPs in case-control group was small.

**Table 2:** Twenty-seven significant SNPs genetic diversity and their Go function annotations.

**Ref_ID**	**SNPs**	**Variants region**	**He**	**Ho**	**PIC**	**Gene locus**
rs47045687	C > T	Intergenic	0.3607	0.25	0.2957	id38041-LYPD6B
rs114843903	C > T	Intergenic	0.5	0.3529	0.375	NYAP2-id50263
rs90835937	C > T	Intergenic	0.4983	0.2941	0.3741	id84954-LOC100138140
rs5881560	T > C	Intron	0.4082	0.3429	0.3249	OSBPL8
rs8678060	T > C	Intron	0.4444	0.3333	0.3457	SYT1
rs37588412	T > C	Intron	0.4444	0.2778	0.3457	LOC786089
rs75762330	C > T	Intron	0.3673	0.303	0.2999	PTK2B^Δ^
rs88640083	A > G	Intergenic	0.1847	0.2059	0.1676	SYK^Δ^-DIRAS2
rs17176625	C > T	Intergenic	0.4824	0.3125	0.3661	LOC-100295568-HTR1B
rs17514753	T > C	Intergenic	0.4775	0.4848	0.3635	TRNAA-UGC-MEI4
rs17518215	C > T	Intergenic	0.4844	0.5294	0.3671	TRNAA-UGC-MEI4
rs22015301	G > C	Downstream	0.405	0.359	0.323	id241467
rs98519900	G > A	Intergenic	0.5	0.2727	0.375	PARK2-PARK2
rs14802054	A > G	Intron	0.4927	0.4545	0.3713	TSC22D1
rs25949166	A > G	Intron	0.4097	0.325	0.3258	PRTFDC1
rs33866959	A > T	Intron	0.4824	0.25	0.3661	CPA6
rs48577224	T > C	Intergenic	0.4965	0.3611	0.3733	NPHP4-LOC100297820
rs49099498	G > A	Intergenic	0.375	0.1842	0.3047	NPHP4-LOC100297820
rs27017918	G > A	Intergenic	0.2945	0.2564	0.2512	LOC100138242-TRNAW-CCA
rs32265465	T > C	Intergenic	0.4244	0.2778	0.3343	LOC789319-CDH11
rs9704351	C > G	Intergenic	0.382	0.4	0.3091	MAP1B-CARTPT
rs20438858	G > A	Intron	0.4284	0.2432	0.3366	TNFRSF21^Δ^
rs22490040	C > T	Intergenic	0.4761	0.3438	0.3628	DEFB112-TFAP2D
rs28580132	A > G	3′-UTR	0.4761	0.2812	0.3628	LOC785873
rs3233588	G > A	Upstream	0.4284	0.4595	0.3366	id550612
rs1988979	G > A	Intron	0.4269	0.2059	0.3358	TBC1D24
rs50888452	G > A	Intron	0.4995	0.3438	0.3748	SRPX2

*He, Heterozygosity Expectation; Ho, Heterozygosity Observation; PIC, Polymorphism Information Content*.

Δ *Three significant SNPs were annotated to the immune-regulated genes, respectively*.

Genetic linkage analysis showed that SNPs rs75762330 was not correlated with rs88640083 (r^2^ = 0.0022) and rs20438858 (r^2^ = 0.043), while rs20438858 was weakly correlated with rs88640083 (r^2^ = 0.22). LD block map showed that rs75762330 was associated with rs77816736, while rs88640083 was associated with rs85927029 and rs85635916, and rs20438858 associated with rs19736020 and rs16711445 (Figure 2B). The SNPs rs77816736, rs85927029, rs85635916, rs19736020, and rs16711445 were annotated to FRMD3, CARD19, IPPK, CYP39A1, and RCAN2, and PTK7 gene, respectively. Although the *P*-values of these five SNPs were >0.05 (Table S6), there was still potential correlation between rs75762330 and FRMD3, rs88640083 and CARD19 and IPPK, rs20438858, and the genes (CYP39A1, RCAN2, and PTK7).

### Independent Sample Population Verification Three Significant SNPs

**Figure 3:**
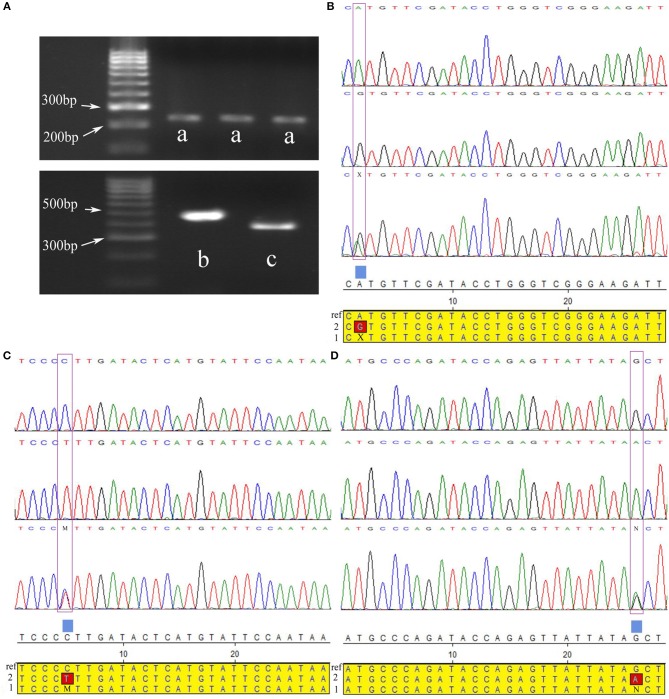
Three significant SNPs were validated for population genetics using direct sequencing in Chinese Holstein cows. **(A)** Gel electrophoresis pattern PCR amplified fragments near three significant SNPs, a-c were PCR amplified fragments of SNPs rs88640083, rs75762330, and rs20438858 regions, respectively. **(B–D)** Directing sequencing results of PCR amplification products near above three important SNPs, and their alignment with reference sequences (ref: reference sequences; 1: heterozygous sequences; 2: variant sequences). The purple boxes were where the three SNPs located. X, M, and N represented the heterozygous types of the three SNPs, respectively.

Correlation analyses were performed on three significant SNPs in another larger independent Chinese Holstein dairy population via direct sequencing (Figure 3). The results showed that the *p*-values of the three SNPs were all < 0.05, indicating that they have significant association with Chinese Holstein mastitis traits (Table 4). The correlation between rs20438858 and risk of mastitis was still statistically significant, with the adjustment allele OR = 0.359 < 1, suggesting that rs20430083 is associated with mastitis susceptibility traits. While, the other two significant SNPs (rs75762330 and rs88640083) with the adjustment allele OR = 2.416 and 1.879 > 1, respectively, were associated with mastitis resistance traits. Population verification data were consistent with the GWAS analysis results. We also noted that AF*e* (Attributable Fraction) value for rs75762330 and rs88640083 was 0.2489 and 0.2426 > 0, respectively, while rs20438858 AF*e* value was −1.786 < 0, also indicating that rs75762330 and rs88640083 are associated with mastitis resistance traits, and rs20430083 is associated with mastitis susceptibility traits.

### Network Analysis of Three Immune-Regulated Genes and Their Proteins

**Figure 4:**
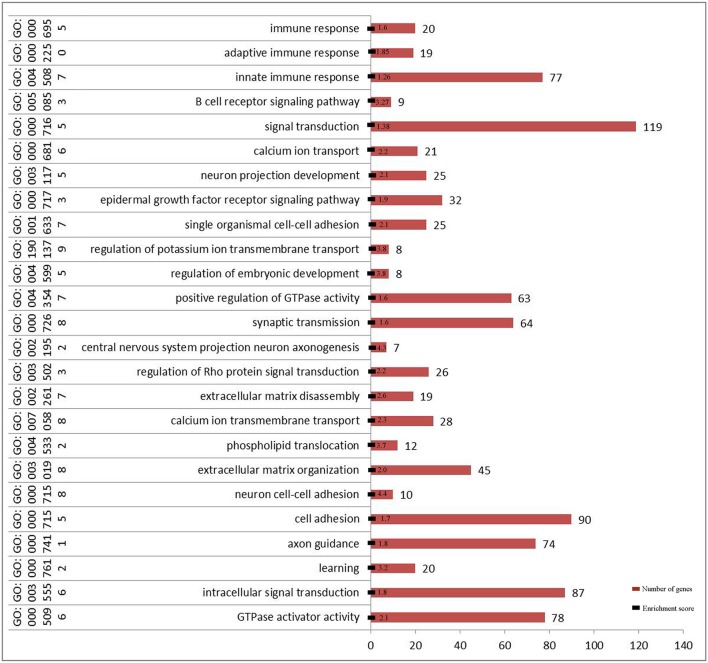
Significant function of GO enrichment analysis (biological process) in 2b-RADseq stage (FDR < 0.001).

Gene ontology enrichment analysis (biological process, cellular component, and molecular function) indicated that three important genes are associated with adaptive or innate immune response in Chinese Holstein (Figure 4 and Figure S4). Kyoto Encyclopedia of Genes and Genomes (KEGG) pathway analysis showed that seven important pathways related to immune response were detected (False Discovery Rate (FDR) < 0.05, Table 3), suggesting that multiple immune response pathways are involved in Chinese Holstein mastitis (Figure S5A). Network protein interaction analysis showed that SYK and PTK2B are involved in natural killer cell mediated cytotoxicity pathway. SYK and AKT1 are in the PI3K-AKT and B cell receptor signaling pathways. PTK2B and AKT1 are in the chemokine signaling pathway. Co-expression scores of the above two proteins are between 0.074 and 0.314 (Figure S5B). TNFRSF21 is involved in the regulation of cytokine-cytokine receptor interaction (FDR = 1.48E-3).

**Table 3:** KEGG enrichment items pathways and regulatory genes of three significant SNPs.

**KEGG ID**	**KEGG term**	**False discovery rate (FDR)**	**Enrichment score**	**Differentially expressed gene (DEG)**
Path:ko04064	NF-kappa B signaling pathway^*^	2.16E-4	1.398	CARD11; CHUK; RELB; MAP3K7; CARD14; TAB1; ATM; SYK; IL1B; CSNK2A1; LBP; TRIM25; PLCG2; IKBKB; CD40; TNFRSF11A; TIRAP; CARD10; DDX58; PRKCB
Path:ko04662	B cell receptor signaling pathway^*^	2.53E-4	1.378	CARD11; DAPP1; CHUK; PIK3CB; GSK3B; PPP3CA; SYK; VAV3; NFATC2; PTPN6; INPP5D; PLCG2; IKBKB; PIK3R2; AKT2; PRKCB
Path:ko04151	PI3K-Akt signaling pathway^*^	5.15E-4	1.311	ITGA8; TNR; THBS1; CDK6; GNB5; PRKAA2; CHUK; ITGB8; GH1; IL2RA; PCK1; FGF13; PDGFA; FGF14; EIF4B; PTK2; CREB1; CDKN1B; PIK3CB; PRKCA; PIK3R5; VEGFC; FN1; PKN1; IL7R; GYS1; EGF; GSK3B; RELN; LAMA4; EFNA3; SYK; LAMB1; FGFR2; ANGPT4; ITGA9; COL4A6; IGF1R; IBSP; MYB; FGF12; CREB5; COL4A2; PGF; COL6A3; RBL2; PHLPP2; RPTOR; IL3RA; LAMA3; LOC104968606; IKBKB; LAMA2; PIK3R2; FLT3; PDPK1; COL9A2; PHLPP1; IFNAR2; AKT2; MET; TNXB; PRLR; ITGA2; LAMC2; PPP2R2B; BCL2L11; PDGFD; THBS4; LPAR1; CSF3R; PPP2CB; LAMA1; LOC530102
Path:ko04650	Natural killer cell mediated cytotoxicity^*^, ^**^	8.26E-4	1.953	LCP2; LOC100850276; SHC3; PTK2B; PIK3CB; PRKCA; CD247; SH2D1B; PPP3CA; SYK; VAV3; NFATC2; PTPN6; PLCG2; PIK3R2; ITGAL; IFNAR2; BRAF; PRKCB
Path:ko04660	T cell receptor signaling pathway	3.02E-3	1.279	CARD11; LCP2; CHUK; MAP3K7; PAK6; PIK3CB; NCK2; CD247; PAK7; GSK3B; PPP3CA; VAV3; NFATC2; PTPN6; IKBKB; PIK3R2; PDPK1; IL5; CD28; AKT2; TEC
Path:ko04062	Chemokine signaling pathway^**^	7.12E-3	1.227	DOCK2; TIAM1; GNB5; SHC3; CHUK; PTK2B; PTK2; ADCY2; PIK3CB; LOC508666; GRK5; CCL5; CCR8; GSK3B; ADCY5; VAV3; STAT5B; PREX1; STAT3; ADCY1; IKBKB; PIK3R2; ELMO1; AKT2; PLCB1; CCR9; BRAF; PARD3; PRKCB
Path:ko04060	Cytokine-cytokine receptor interaction^***^	9.15E-3	1.119	IL12RB2; IL18; GH1; IL2RA; PDGFA; IFNE; IL1RAP; LOC508666; IL20RA; IL12RB1; VEGFC; TNFSF18; RELT; IL7R; CCL5; IFNL3; CCR8; EGF; IL17C; IL1B; IL1R2; IL18R1; IL3RA; FLT3; CD40; TNFRSF11A; TNFRSF21; BMPR1A; IL5; IFNAR2; IL17B; CCR9; MET; PRLR; IL10RA; ACVR1; PDGFD; IL13RA1; CSF3R

* *SYK gene is involved in KEGG enrichment pathway*.

** *PTK2B gene is involved in KEGG enrichment pathway*.

*** *TNFRSF21 gene is involved in KEGG enrichment pathway*.

**Table 4:** Population genetics validated three significant SNPs in another individual population via case-control study.

**SNPs**	**Base**	**Case**	**Control**	AFe^*^	OR^**^	**χ^2^**	**P < **	**Df =**
rs88640083	G	47 (a)	152 (b)	0.2426	1.879	5.578	0.025	1
	A	26 (c)	158 (d)					
rs75762330	T	36 (a)	89 (b)	0.2489	2.416	10.279	0.005	1
	C	37 (c)	221 (d)					
rs20438858	T	17 (a)	142 (b)	−1.786	0.359	12.34	0.001	1
	C	56 (c)	168 (d)					

* *AF_e_ refers to the proportion of mastitis caused by the SNPs to all mastitis; AFe > 0 indicating that the SNPs positive associated with mastitis susceptibility, while AFe < 0 is negative*.

** *OR is the exposure ratio (Odds ratio); OR > 1 indicating that the SNPs positive associated with mastitis susceptibility, while OR < 1 is negative*.

### Expression Variation of PTK2B, SYK, TNFRSF21, TLR4, AKT1, NF-κB, IL-1β, IL-8, and IL-10 in Case-Control Group

**Figure 5:**
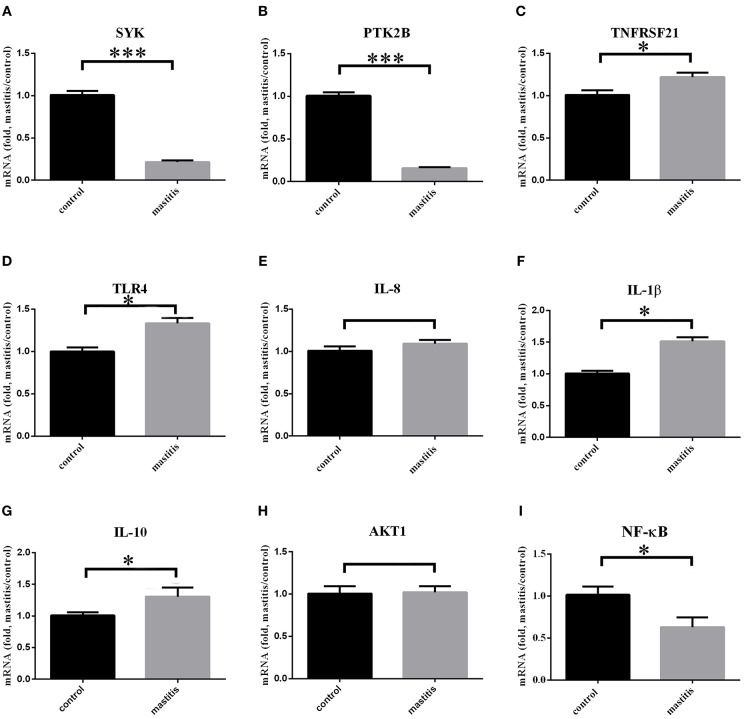
Three immune-related candidate genes, TLR4, AKT1, NF-κB, IL-1β, IL-8, and IL-10 mRNA expression levels in peripheral blood leukocytes of experimental cows. ^***^Represent *P* < 0.001. ^*^Represent *P* < 0.05. **(A–I)** Immune-related gene expression in mastitis-control cows.

Total of nine genes (three candidate genes: SYK, PTK2B, and TNFRSF21; TLR4, Toll-like receptor; AKT1, Protein kinase B; NF-κB, Nuclear factor-kappa B; IL-8 and IL-1β, pro-inflammatory factor; IL-10, inflammation and immunosuppressive factor) were selected for qRT-PCR analsysis (Figure 5). It showed that expression of SYK and PTK2B was down-regulated significantly (*P* < 0.001) in mastitis cows in comparison to the control group, while TNFRSF21 was up-regulated. AKT1 expression level had no significant difference (*P* > 0.05), while NF-κB down-regulated (*P* < 0.05). TLR4 (*P* < 0.05) and two other pro-inflammatory factors (IL-8, *P* > 0.05; IL-1β, *P* < 0.01) were also up-regulated. However, IL-8 expression level had no significant difference in peripheral white blood cells (*P* > 0.05). The expression level of IL-10 were up-regulated (*P* < 0.05). The mean C_t_ value with SD of the β-actin in case-control samples were 20 ± 0.2665 and 20.43 ± 0.2789, respectively (Figure S7A).

### Expression Variation of PTK2B, SYK, TNFRSF21, TLR4, AKT1, NF-κB, IL-8, IL-1β, and IL-10 in bMECs via LPS Stimulation

**Figure 6:**
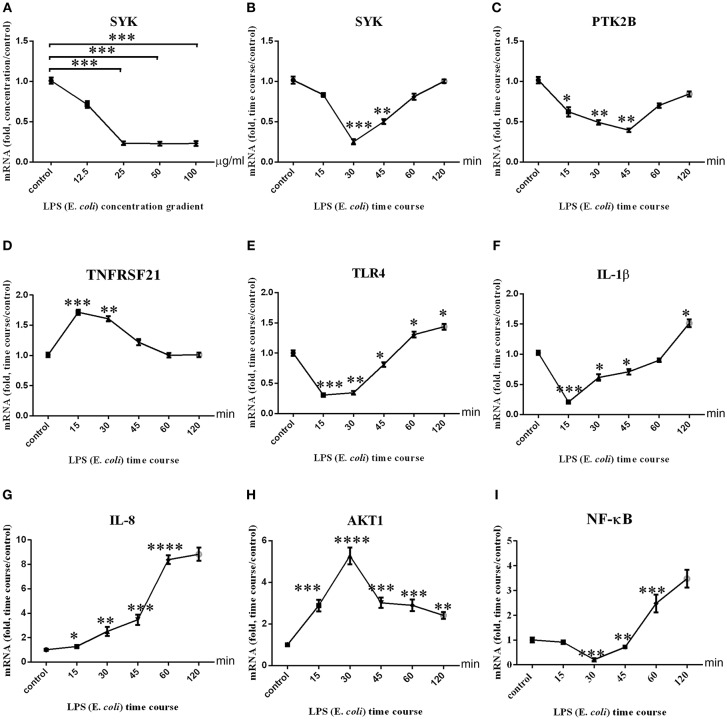
Three immune-related candidate genes, TLR4, AKT1, NF-κB, IL-1β, IL-8, and mRNA expression levels in bMECs. **(A)** The mRNA expression level of SYK after stimulation of BMSCs by LPS concentration gradient. **(B–I)** The mRNA expression levels of SYK, PTK2B, TNFRSF21, TLR4, AKT1, IL-1β, and IL-8 after stimulation of bMECs by LPS (*E. coli*, 25 μg/ml) time courses. ^****^represent *P* < 0.0001. ^***^represent *P* < 0.001. ^**^represent *P* < 0.01. ^*^represent *P* < 0.05.

The expression level of candidate genes and related pro-inflammatory factors was detected in LPS-stimulated bMECs *in vitro*. The optimal LPS concentration was 25 μg/ml (Figure 6A). We then performed the time course experiment with this concentration (Figures 6B–I). Figure 6B showed that SYK expression was down-regulated, and then rebounded from 30 to 120 min. PTK2B expression level was down-regulated at 0–45 min, and then up from 45 to 120 min (Figure 6C). TNFRSF21 expression level was up-regulated from 15 to 60 min, and then down during 60–120 min (Figure 6D). The expression of TLR4 was down-regulated in 0–45 min and up-regulated in 60–120 min (Figure 6E). IL-1β expression levels were down-regulated within 0–60 min and up-regulated at the 120 min (Figure 6F). IL-8 expression up-regulated and had an upward trend, which was almost the same at 60–120 min (Figure 6G). AKT1 expression was up-regulated, and showed an upward trend at 0–30 min and a downward trend at 30–120 min (Figure 6H). NF-κB expression was down-regulated within 0–45 min, while up-regulated after 60 min (Figure 6I). The expression of IL-10 was up-regulated, and showed an upward trend at 0–15 and 30–45 min and a downward trend at 15–30 and 45–120 min (Figure S6). The mean C_t_ value with SD of the β-actin in LPS concentration stimulated and time course were shown in Figures S7C,D, respectively.

## Discussion

Clinical mastitis is a common unavoidable disease that seriously affects the health benefits and breeding profits of dairy cows (3, 38). In the production process, mastitis is still not fully controlled despite thorough hygiene, antibiotic treatment, vaccination, and other measures (52). Therefore, the association between dairy cows' genetic variation and mastitis is receiving more and more attention (18, 35, 53, 54). This study sought to screen for significant differential SNPs associated with clinical mastitis via GWAS-2bRADseq, to identify potential clinical mastitis susceptibility or resistance genes and to verify for their authenticity and role in mastitis resistance/tolerance *in vitro* and *in vivo*.

Two-stage correlation analysis for three mastitis significant SNPs and Population genetic verification.

In stage I, a total of 10,058 high quality SNPs were screened by 2b-RADseq. The average LD coefficient of 100 kb on the genome of case-control two Chinese Holstein cows was about 0.5. However, the corresponding LD coefficient was still above 0.3 when the distance was 1,000 kb. It is suggested that the genetic diversity of the population of Chinese Holstein cows decreased after acclimatization and selection, and the linkage degree (LD) between the loci were strengthened. Bayesian model screened out 42 significant SNPs when *P* < 0.001, while the Logical regression analysis model had no SNPs; however, the Bayesian model had 257 significant SNPs when *P* < 0.01, while Logical regression analysis model labeled 51 significant SNPs. Therefore, the accuracy of molecular breeding value (MBV) prediction is important for the successful application of genome selection (GS) (55). Therefore, selecting the appropriate analytical model based on genotypic and phenotypic data is critical to achieving accurate results (56). This study, two association analysis models (Bayesian model and logical regression model) were used to process 2b-RAD sequencing data to improve accuracy and reduce false positives. It's obvious that the thresholds values of the two analytical models were significantly different. Giving us hints that when processing data, it must be statistically analyzed according to their respective thresholds. Comparison of results from two analytical models, we reduced the dimensions when processing data and only considered the SNPs associated with immune-related genes and their signaling pathways. Finally, we identified three (rs75762330, rs88640083, and rs20438858) novel bovine mastitis traits significant SNPs in Chinese Holstein cows.

With regarding to stage II, we used a case-control study to verify the association between three significant SNPs and bovine mastitis. We compared the exposure ratios of important SNPs in case and control groups, and only considered the relationship between SNP and mastitis traits under the exclusion of external matching factors (57–59). According to the Pitman efficiency increment formula (2R/(R+1)), we determined the appropriate sample size and gained higher test efficiency (1/4). Here, we validated the association of three important SNPs with cows mastitis. SNP rs75762330 and rs88640083 (OR > 1, AF*e* > 0) are positive, and SNP rs20438858 is negative (OR < 1, AF*e* < 0) associated with mastitis susceptibility, respectively.

Three significant mastitis-associated SNPs are located in genomic non-coding sequences and in low or moderate genetic polymorphisms.

Previous researchers found that conserved non-coding regions (CNCs) in introns and near genes show large allelic frequency shifts, similar in magnitude to missense variations, suggesting that CNCs are critical for gene function regulation and evolution in many species, including yeast, fruit flies, and vertebrates (60–64). Our GWAS data provided a statistical list of SNPs associated with mastitis traits in dairy cows, where three significant SNPs (rs75762330, rs88640083, and rs20438858) are annotated to immune-related genes are located in non-coding regions (intron and intergenic) of the genome. GWAS studies also showed that the mastitis traits were controlled by multiple loci in the genome, and the genetic effects of each locus were relatively small (18, 26, 34, 38). SNP rs88640083 (PIC = 0.1676 < 0.25) had low polymorphism, while SNPs rs75762330 (0.25 < PIC = 0.2999 < 0.5) and rs20438858 (0.25 < PIC = 0.3366 < 0.5) were moderately polymorphic. Despite the low or moderately heritability of clinical mastitis, identifying significant SNPs associated with clinical mastitis are extremely important for breeding programs that reduce the incidence of mastitis (36, 65).

### LPS Stimulates bMEC in a Dose-and Time-Dependent Manner *in vitro*

Pathogen-specific mastitis traits are direct indicators of cow mastitis infection. *E. coli* is the main pathogen of clinical mastitis, while *Staphylococcus aureus* and *Streptococcus uberis* mainly cause sub-clinical mastitis (8, 66). Once infected, mammary gland innate immunity is initiated; bMECs act as the first line of defense against exogenous pathogens and secrete several cytokines (e.g., IL-1β, IL-8, and TNF-α) to recruit neutrophils into the mammary gland (8, 67, 68), causing a sharp rise in SCC intra-mammary infection in early- and late-lactation (69–71) and the corresponding mastitis phenotype. Therefore, bMEC is the key for rapid elimination of bacteria and prevention of mastitis (inflammation of bovine mammary gland) (72). Exogenous stimulation of bMEC activity was dose- and time-dependent manner as well (73–75). SYK is a non-receptor tyrosine kinase that plays an essential role in various biological functions (76). SYK-Pyk2 (PTK2B) axis is participated in regulation of TNF activation in human neutrophils (77). Therefore, the optimal LPS stimulation concentration and time was determined with reference to the SYK expression level in bMECs. Then we counted and compared the changes of TLR4, IL-1β, IL-8, and NF-κB expression in LPS-stimulated bMECs time course, and their expected parameters and fold differences serve as positive controls (67), and confirming the reliability of time course bMECs study results: the down-regulation of PTKTB2 and SYK, respectively, and the up-regulation of TNFRSF21.

### Three Important Immune-Regulated Genes (PTK2B, SYK, and TNRSF21) Associated With Mastitis Traits in Chinese Holstein Cows

Gene Ontology function analysis annotated three significant SNPs to three immune regulatory genes PTK2B, SYK, and TNFRSF21, respectively. The LD block data also showed that there were strong LD relationship between rs75762330 and PTK2B, rs88640083 and SYK, rs20438858 and TNFRSF21, suggesting that these three immune-regulated genes are important candidate genes associated with mastitis traits in Chinese Holstein cows.

PTK2B is a protein tyrosine kinase that regulates humoral immune response and homeostasis of innate immune cells (78–81). It is involved in regulating TLR4 cascade and IL-10 production in macrophages and affects the migration of dendritic cells (DCs) (78, 80, 82). PTK2B participates in the innate immune response through interaction with IkappaB kinase β (IKKβ) and TANK-binding kinase 1 (TBK 1) (83), and is critical for neutrophil degranulation and host defense against bacterial infection (84). PTK2B expression levels in bPBLs of mastitis cows were significantly down-regulated (*P* < 0.001) and had a time course difference in LPS-stimulated bEMCs, suggesting that PTK2B plays an important role in clinical mastitis.

SYK is an important regulator factor for TLR4 signaling pathway (85, 86), and regulates the secretion of IL-1β through CARD 9 in dendritic cells (87). In macrophages, SYK, as a downstream regulatory molecule of TLR4 and IL10, plays a dual role in pro-inflammatory and anti-inflammatory responses (88, 89). SYK is also involved in regulating the proliferation of dairy mammary epithelial cells, milking cycles, and milk production (90). The expression levels of SYK in bPBLs and LPS-stimulated bMECs of mastitis cows were significantly down-regulated, indicating that SYK is involved in inflammatory and immune response of clinical mastitis. As shown in Figures 6B,C, the temporal sequences of SYK, PTK2B, TLR4, and IL-1β expression are similar, suggesting that there might be an immune response pathway in bMECs participating in bovine mastitis inflammatory response. However, as the stimulation is prolonged, TLR4, NF-κB, and IL1β are up-regulation at later time points, suggesting that different immune response mechanisms may be initiated, and the phenomenon which needs further study.

TNFRSF21 is a member of the TNF/TNFR family, and plays a critical role in immune response and inflammation (91–93). It regulates the degeneration of the mammary gland and providing protection against infection (94). TNFRSF21 is also involves in NF-κB signaling pathway (95), which is the key pathways to identify exogenous pathogens and induce inflammation and immune response. TNFRSF21 was significantly up-regulated in bPBLs of mastitis cows and LPS-stimulated bMECs, indicating that TNFRSF21 plays an important role in bovine mastitis inflammation (96).

### Three Immune-Regulated Genes (PTK2B, SYK, and TNFRSF21) Initiate Different Immune Response in bPBLs and bMECs to Cope With Mastitis

**Figure 7:**
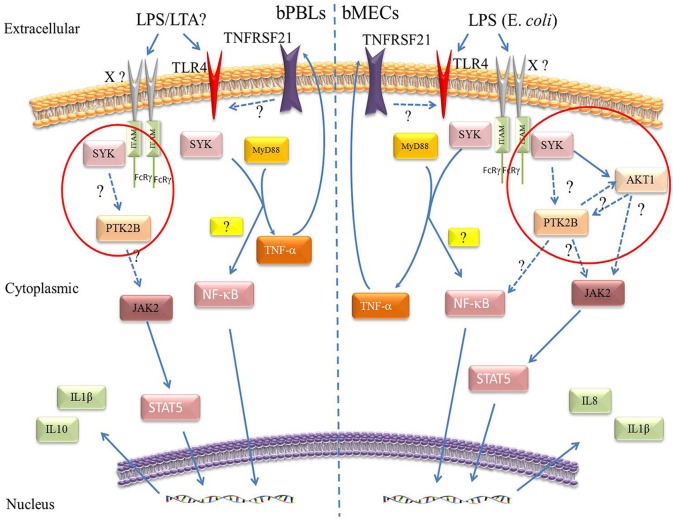
Molecular mechanisms prediction of the three immune-regulated genes in Chinese Holstein cows. Peripheral blood leukocytes of clinical mastitis cows **(Left)** and LPS (*E. coli*) stimulated bMECs **(Right)**. Solid arrows represent known mechanisms and dashed arrows represent unknowns.

In bPBLs and bMECs, PTK2B, SYK, and TNFRSF21 are involved in different mechanisms of immune responses associated with mastitis (Figure 7). There was no significant difference in the expression level of AKT1 in bPBLs. But interestingly, they were significantly up-regulated in bMECs, and the trends were completely opposite to SYK and PTK2B, suggesting that PTK2B, SYK, and AKT1 participate in the immune cascade response of bMECs. PTK2B-AKT1 is involved in chemokine signaling pathway, while SYK-AKT1 is involved in B cell receptor signaling pathway and PI3K-AKT signaling pathway. However, AKT1, as a key player in the Jak2/STAT5 pathway, plays an important role in the regulation of differentiation, secretion, survival, and proliferation of mammary epithelial cells as well as mammary remodeling and lactation sustainability in dairy cows (90, 97–100). JAK2 and STAT5, two key players in the immune response, are associated with cow mastitis susceptibility (11, 101). Mastitis caused by bacterial infection of the mammary gland induces IL8 expression (102), and IL-8 plays an important role in the recruitment and degranulation of neutrophils in mammary gland (13). However, there were no significantly different in the expression levels of IL-8 in bPBLs of Chinese Holstein, while there were significant differences in LPS-stimulated bMECs, indicating that IL-8 has vital function on innate immunity of bMECs. The expression of IL-1β was significantly up-regulated in both clinical mastitis bPBLs and LPS-stimulated bMECs, suggesting that it is essential in bovine mastitis. IL-10, an anti-inflammatory cytokine involved in adaptive and innate immune regulation, plays a critical role in infection by limiting excess immune response to the pathogens and preventing host damage (103–106). It's regulated by Pyk2 (PTK2B) in LPS-stimulated mouse macrophages, and negatively regulated by SYK in dendritic cells (82, 89). Therefore, up-regulation of IL-10 expression in bPBLs, suggesting that IL-10 may be regulated by PTK2B and SYK, and thus participate in the regulation of inflammatory response in dairy cow mastitis. NF-κB pathway plays a central role in the host innate and acquired immune responses to microbial pathogen infection and is essential for maintaining immune homeostasis (107, 108). NF-κB expression was down-regulated in mastitis cows' bPBLs and time-dependent in LPS-stimulated bMECs. Therefore, we hypothesized that SYK and TNFRSF21 may be involved in the NF-κB pathway, stimulating IL-1β secretion and promote mastitis inflammation in both bPBLs and bMECs. SYK and PTK2B may be involved in the SYK/PTK2B/AKT1/JAK2 pathway in bMECs that stimulates IL-8 secretion and recruits neutrophils to kill pathogenic microorganisms, resulting in the resistance to mastitis inflammation.

## Conclusions

In this study, we were committed to improve understanding of biogenetic variation of mastitis, and providing a genetic basis for constructing and improving anti-mastitis characteristic in Chinese Holstein cows. First, we used GWAS-2b-RADseq to identify significant SNPs. We used two-stage correlation analysis to find 27 significant SNPs associated with risk of mastitis, among which three SNPs (rs75762330, rs88640083, and rs20438858) were annotated to immune-regulated genes (PTK2B, SYK, and TNFRSF21). Next, population genetic verification of three important SNPs was performed in another individual cow population, confirming that SNPs rs75762330, rs88640083, and rs20438858 were associated with mastitis susceptibility. Transcriptional analysis showed that PTK2B and SYK expression level was down-regulated in both bPBLs of clinical mastitis cows and *in vitro* LPS (*E. coli*)–stimulated bMECs indicating that PTK2B and SYK are important candidate genes associated with mastitis resistance traits. TNFRSF21 was up-regulated suggesting that TNFRSF21 is an important candidate gene associated with mastitis susceptibility traits. Network analysis showed that different immune response mechanisms are activated to deal with pathogen (*E. coli*) invasion. However, their molecular mechanisms for response to bovine mastitis remain unclear. Do SYK and TNFRSF21 participate in the NF-κB pathway? What are their statuses in bMECs? What are the co-expression mechanisms of SYK and PTK2B in bMECs in response to pathogens? Is there a SYK/PTK2B/AKT1/JAK2 pathway in bMECs or bovine mammary gland? Answering these questions will keep scientists interested in the immune response mechanisms caused by bovine mastitis.

## Data Availability

The publicly available datasets generated for this study can be found in the NCBI SRA database - PRJNA556499 - https://www.ncbi.nlm.nih.gov/sra/PRJNA556499.

## Ethics Statement

The procedures involving animals were approved by the Animal Welfare Committee of Nanjing Agricultural University, Nanjing, China, and approval No.20160615. And all animal experiments were carried out in strict accordance with the guidelines and rules established by the committee.

## Author Contributions

YC, JL, HL, and GW co-designed the experiment. FY, FC, LL, LY, TB, CL, DY, MZ, XJ, JL, and LY participated in the screening of experimental animals and blood sample extraction. FY and FC sorted and analyzed 2b-RAD sequencing data. FY wrote and calibrated experiments manuscripts. The final manuscript was read and authorized by all authors.

### Conflict of Interest Statement

The authors declare that the research was conducted in the absence of any commercial or financial relationships that could be construed as a potential conflict of interest.

## Supplementary Material

Figure S1 Whole genome-wide sequencing (2B-RADseq) diagram of Chinese Holstein cows.

Figure S2 Distribution of SNP markers on chromosomes: the horizontal axis indicated the coordinates of the physical position of the chromosomes; the vertical axis represented the number of the corresponding SNPs (window size, 20 Kbp; Step length, 10 Kbp).

Figure S3 Genotyping of all SNPs and evaluation of two GWAS analysis models. (1) Genotyping and genotype score of 10058 SNPs **(A)**: 0 (two bases of the type were different from the reference genome); 1 (two bases of typing were the same as one of the reference genomes); 2 (two bases of typing were the same as those of the reference genome). (2) The consistency of Bayesian **(B)** and logistic regression **(C)** analysis for SNPs observed and expected value –log10 (P), respectively. The *P*-value observation is almost the same as the expected, indicated that the analysis model was reasonable. There were several SNPs *P*-values exceeded expected, which suggested that these locus might be significantly associated with dairy cows' mastitis traits.

Figure S4 Hierarchical Network of candidate Gene function based on go enrichment Analysis. Each circle represented a Go entry; the color indicated the enrichment degree, the deeper the color (yellow), the more genes enriched in the Go entry; the direction of the arrow indicated hierarchic relationship.

Figure S5 Candidate genes interaction network Diagram based on KEGG Database. **(A)** Protein-protein interaction (93 genes) network map constructed in STRING 10.5 with the three candidate genes as the core (avg. local clustering coefficient: 0.707). **(B)** Based on co-expression of homologous genes from other species (co-expression scores: 0.074–0.314), predicted association between SYK and PTK2B.

Figure S6 The mRNA expression levels of IL-10 after stimulation of bMECs by LPS (*E. coli*, 25 μg/ml) time course.

Figure S7 The mean C_t_ value with SD of the β-actin in bPBLs of case-control samples **(A)** and bMECs **(C,D)**, and identification of bMECs by fluorescence confocal microscope [**(B)**, 1: DAPI staining of cell nucleus, 2: immunofluorescence staining of Cytokeratin 18, 3: synthetic map of nuclear (1) and cytoplasmic (2) staining].

Table S1 Illumina base recognition and mass value correspondence table.

Table S2 The top five principal component data of all SNPs for each sample.

Table S3 Three genotypic risk estimation data of case-control by Armitage-test.

Table S4 Forty two important SNPs screened out via Bayesian Analysis Model.

Table S5 The logistic regression model screened out 51 important SNPs.

Table S6 Five SNPs associated with three important SNPs screened by the Bayesian Model and Logical regression analysis model.

Table S7 Three pairs of PCR amplification primers for population genetics validation.

Table S8 Primers of internal reference gene (β-actin), three immune-regulate genes, TLR4, AKT1, NF-κB, and three interleukin genes for RT-qPCR analysis.

Table S9 Fst values of 27 significantly different SNPs.

Data Sheet 1 A supplement to the “Materials and Methods” section.
