# Supplementary figures and images for: Three Novel Players: PTK2B, SYK, and TNFRSF21 Were Identified to Be Involved in the Regulation of Bovine Mastitis Susceptibility via GWAS and Post-transcriptional Analysis
Source: Front Immunol. 2019 Aug 6;10:1579. doi: 10.3389/fimmu.2019.01579 (PMC6691815; doi:10.3389/fimmu.2019.01579)

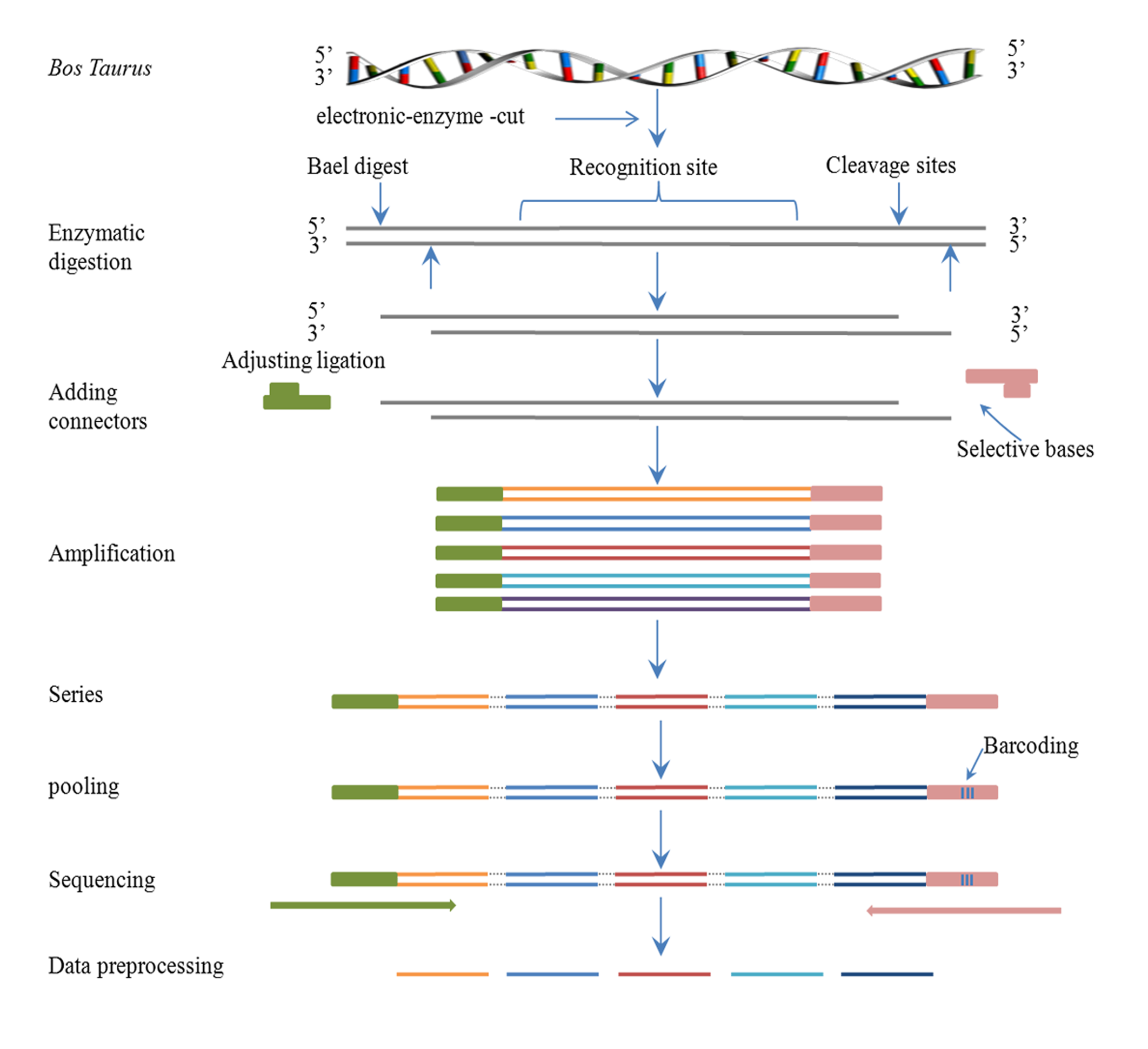

Supplement: Figure S1 — Whole genome-wide sequencing (2B-RADseq) diagram of Chinese Holstein cows. [file Image_1.TIF]

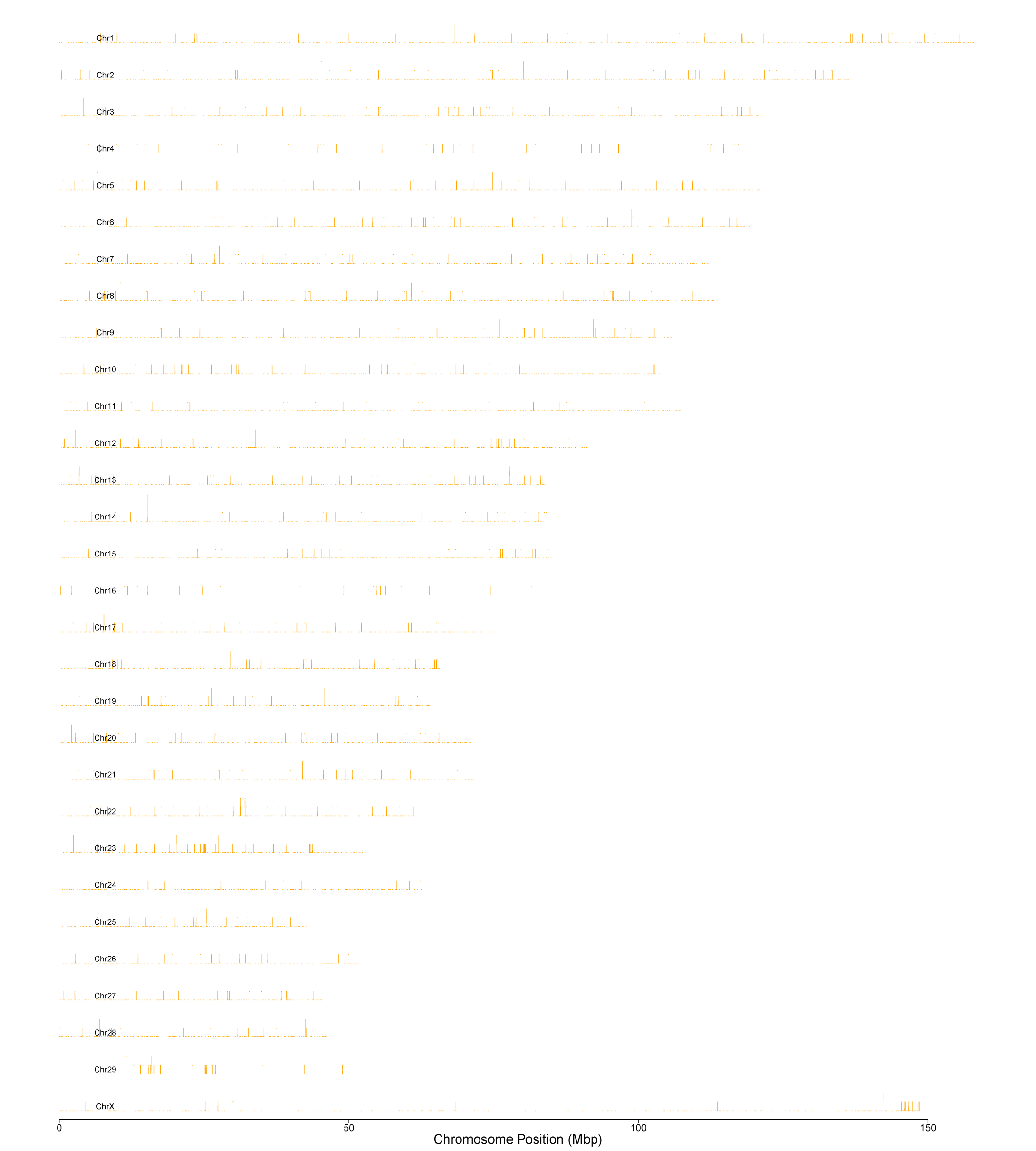

Supplement: Figure S2 — Distribution of SNP markers on chromosomes: the horizontal axis indicated the coordinates of the physical position of the chromosomes; the vertical axis represented the number of the corresponding SNPs (window size, 20 Kbp; Step length, 10 Kbp). [file Image_2.TIF]

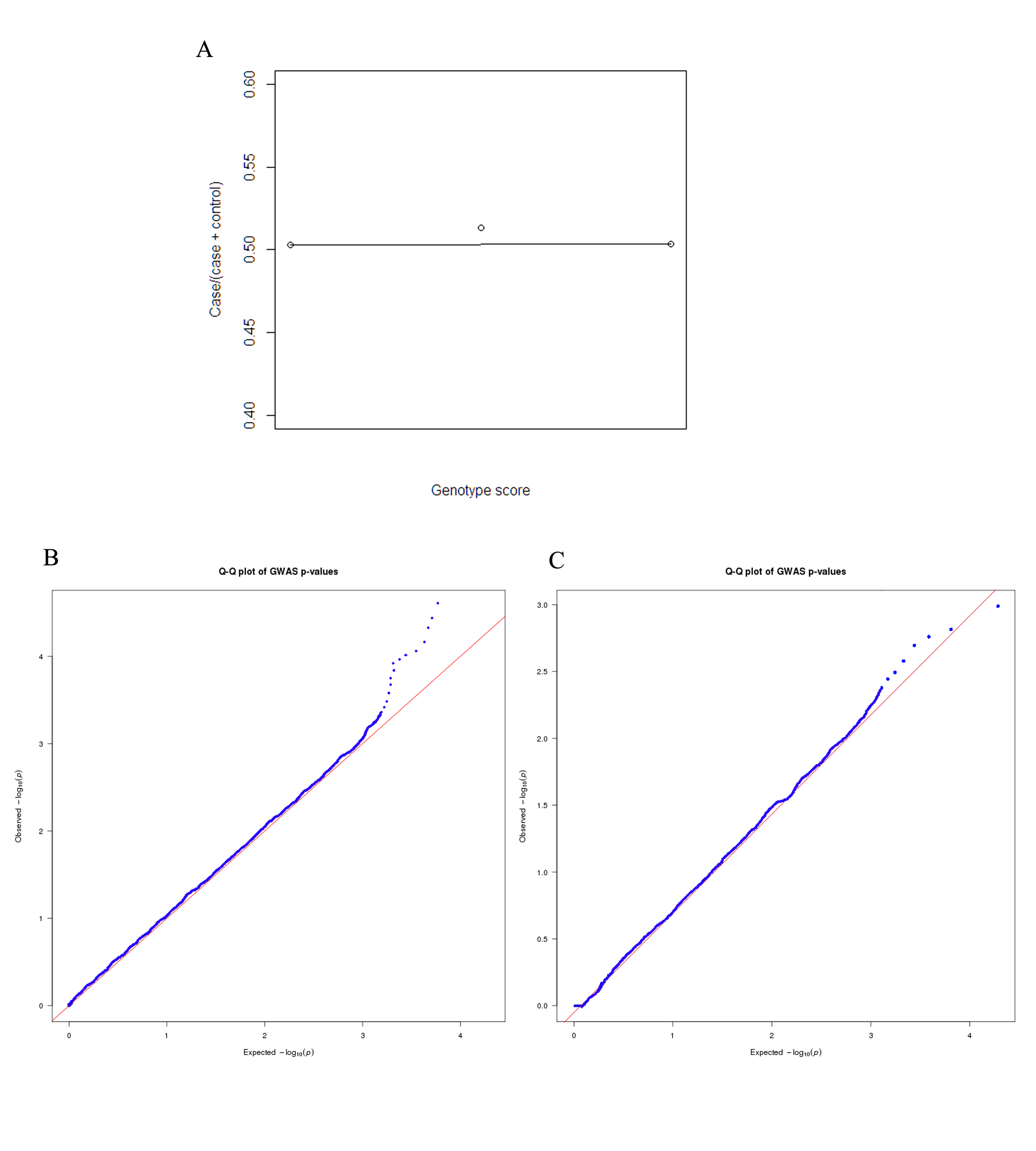

Supplement: Figure S3 — Genotyping of all SNPs and evaluation of two GWAS analysis models. (1) Genotyping and genotype score of 10058 SNPs (A): 0 (two bases of the type were different from the reference genome); 1 (two bases of typing were the same as one of the reference genomes); 2 (two bases of typing were the same as those of the reference genome). (2) The consistency of Bayesian (B) and logistic regression (C) analysis for SNPs observed and expected value –log10 (P), respectively. The P-value observation is almost the same as the expected, indicated that the analysis model was reasonable. There were several SNPs P-values exceeded expected, which suggested that these locus might be significantly associated with dairy cows' mastitis traits. [file Image_3.TIF]

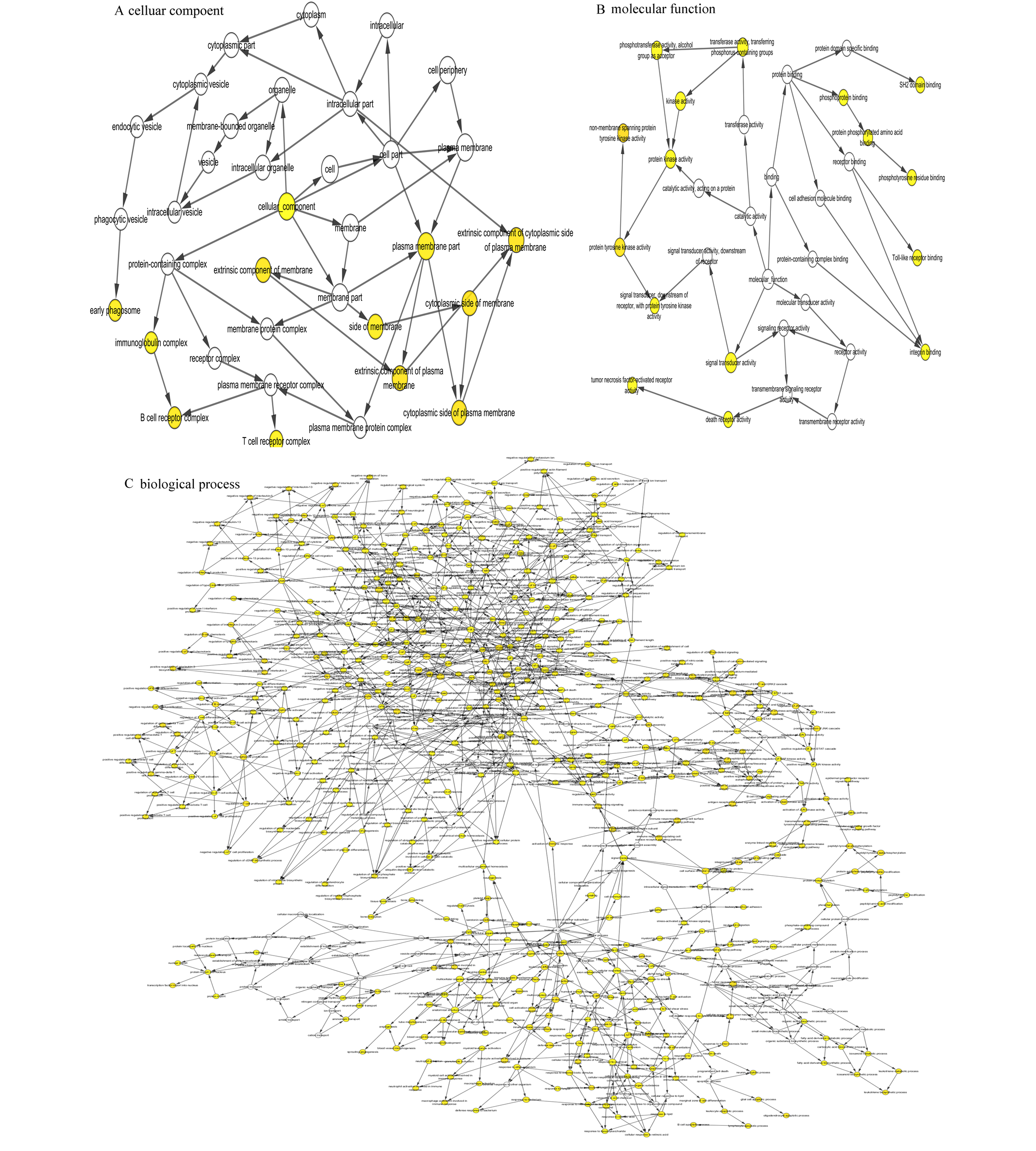

Supplement: Figure S4 — Hierarchical Network of candidate Gene function based on go enrichment Analysis. Each circle represented a Go entry; the color indicated the enrichment degree, the deeper the color (yellow), the more genes enriched in the Go entry; the direction of the arrow indicated hierarchic relationship. [file Image_4.TIF]

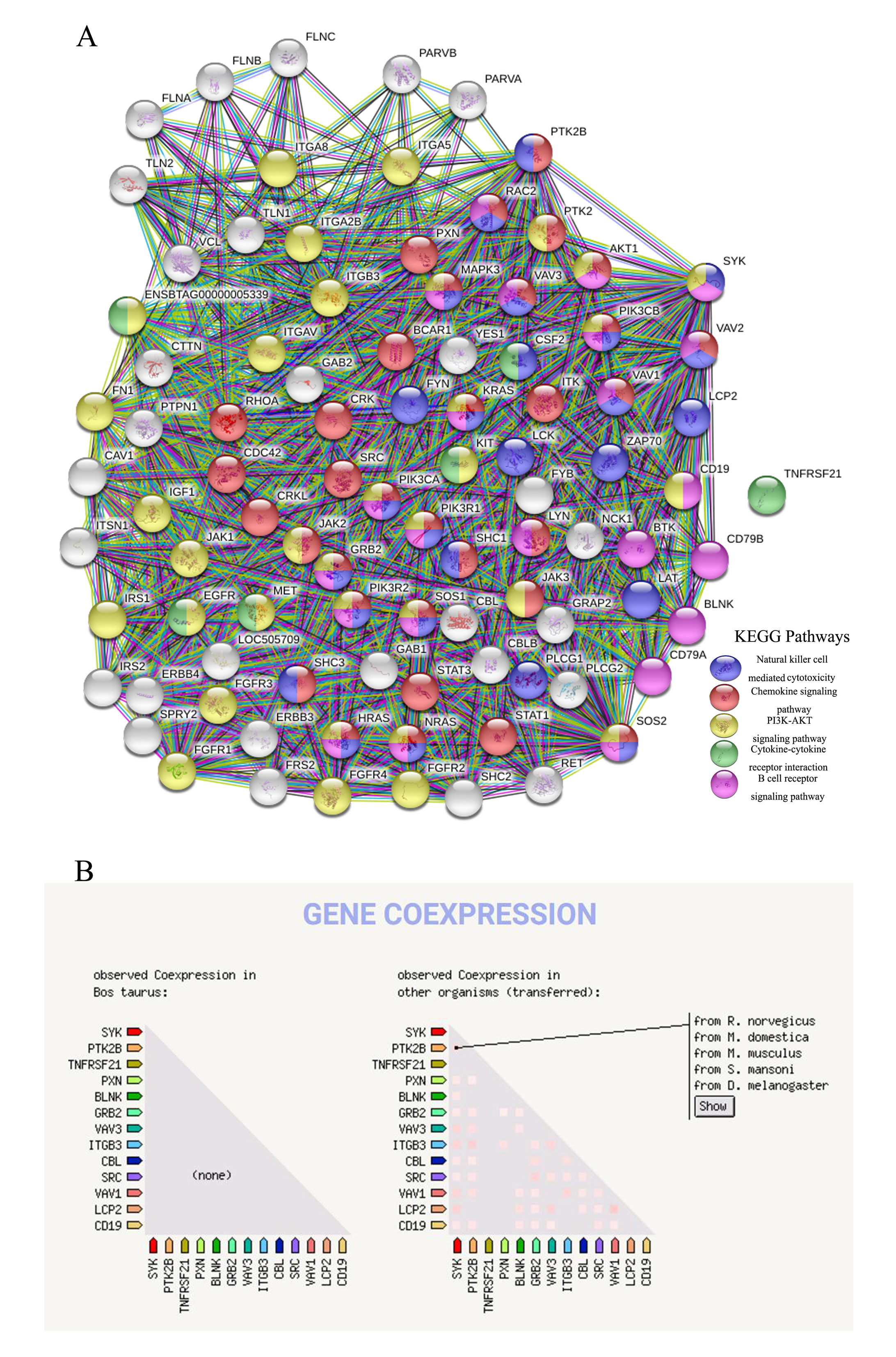

Supplement: Figure S5 — Candidate genes interaction network Diagram based on KEGG Database. (A) Protein-protein interaction (93 genes) network map constructed in STRING 10.5 with the three candidate genes as the core (avg. local clustering coefficient: 0.707). (B) Based on co-expression of homologous genes from other species (co-expression scores: 0.074–0.314), predicted association between SYK and PTK2B. [file Image_5.TIF]

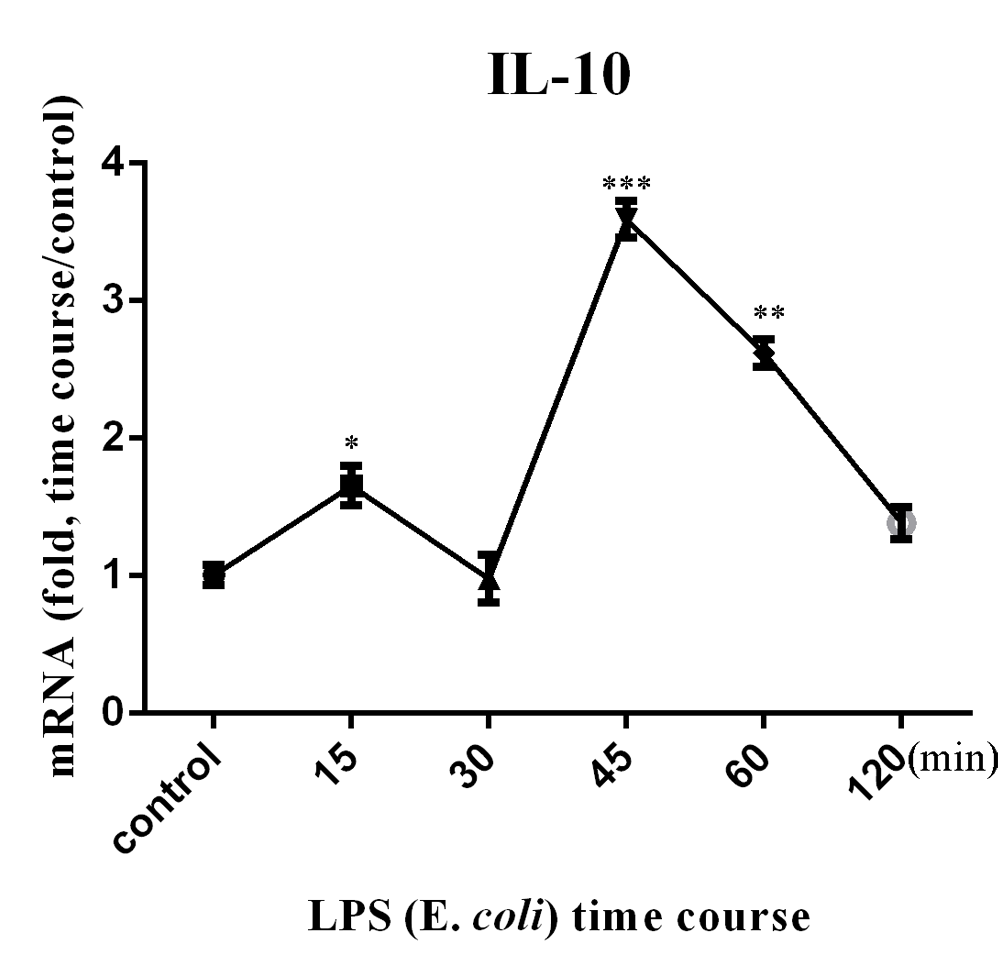

Supplement: Figure S6 — The mRNA expression levels of IL-10 after stimulation of bMECs by LPS (E. coli, 25 μg/ml) time course. [file Image_6.TIF]

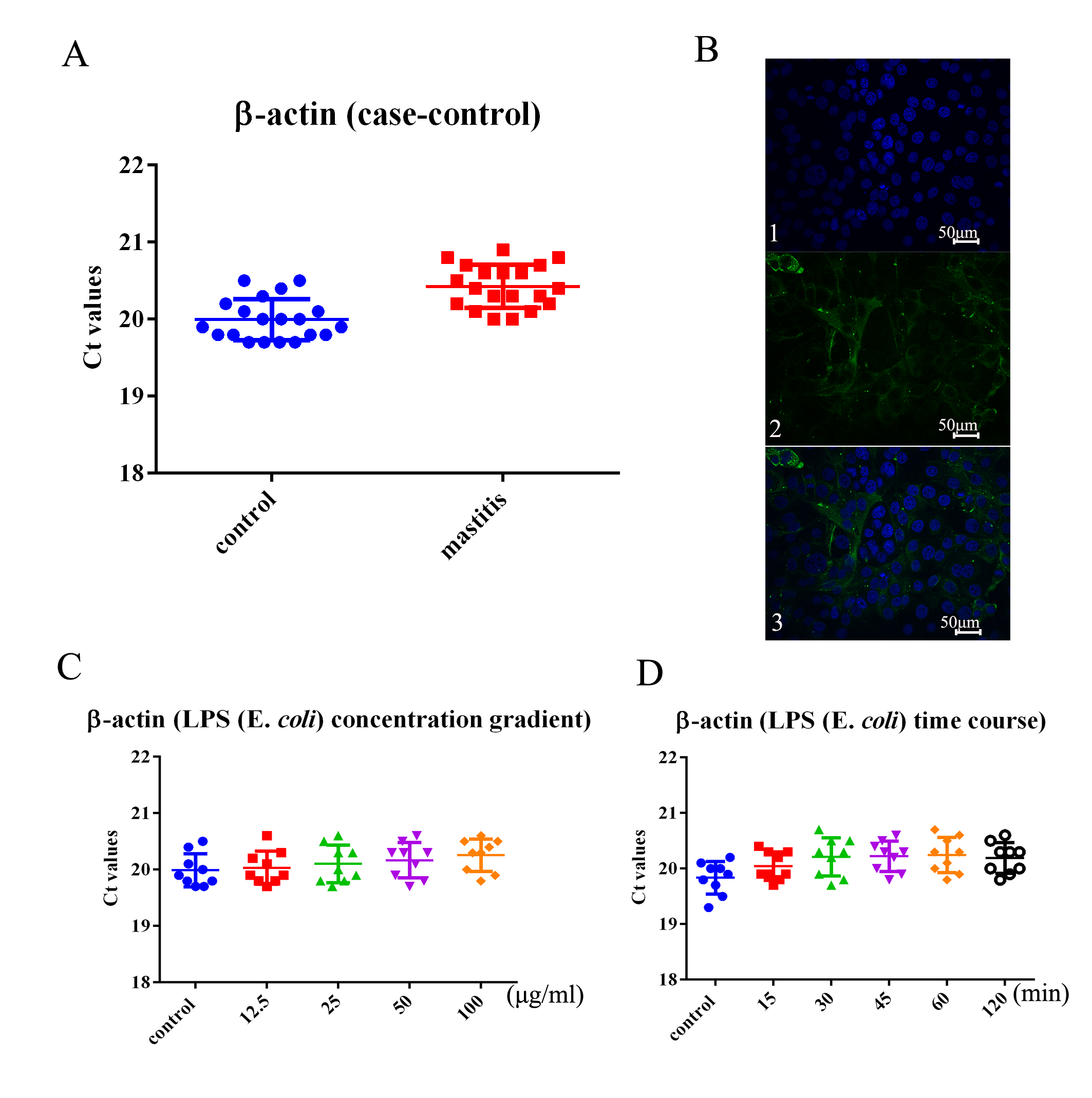

Supplement: Figure S7 — The mean Ct value with SD of the β-actin in bPBLs of case-control samples (A) and bMECs (C,D), and identification of bMECs by fluorescence confocal microscope [(B), 1: DAPI staining of cell nucleus, 2: immunofluorescence staining of Cytokeratin 18, 3: synthetic map of nuclear (1) and cytoplasmic (2) staining]. [file Image_7.TIF]
