# Supplementary material for: Three Novel Players: PTK2B, SYK, and TNFRSF21 Were Identified to Be Involved in the Regulation of Bovine Mastitis Susceptibility via GWAS and Post-transcriptional Analysis
Source: Front Immunol. 2019 Aug 6;10:1579. doi: 10.3389/fimmu.2019.01579 (PMC6691815; doi:10.3389/fimmu.2019.01579)
Supplement: Table S1 — Illumina base recognition and mass value correspondence table. [file Table_1.DOCX]

| Phred score | Base recognition error rate | Base recognition accuracy | Q-score |
| --- | --- | --- | --- |
| 10 | 1/10 | 90% | Q10 |
| 20 | 1/100 | 99% | Q20 |
| 30 | 1/1000 | 99.9% | Q30 |
| 40 | 1/10000 | 99.99% | Q40 |
