# Supplementary material for: Three Novel Players: PTK2B, SYK, and TNFRSF21 Were Identified to Be Involved in the Regulation of Bovine Mastitis Susceptibility via GWAS and Post-transcriptional Analysis
Source: Front Immunol. 2019 Aug 6;10:1579. doi: 10.3389/fimmu.2019.01579 (PMC6691815; doi:10.3389/fimmu.2019.01579)
Supplement: Table S2 — The top five principal component data of all SNPs for each sample. [file Table_2.DOCX]

| Group | Sample | PC1 | PC2 | PC3 | PC4 | PC5 |
| --- | --- | --- | --- | --- | --- | --- |
| case | 1 | -0.0941 | 0.028066 | -0.15007 | 0.123898 | -0.34395 |
| case | 2 | 0.202894 | -0.04839 | 0.052181 | -0.03413 | 0.055241 |
| case | 3 | -0.10136 | 0.23241 | -0.13033 | -0.02663 | 0.029933 |
| case | 4 | 0.183967 | -0.05688 | 0.029148 | -0.0154 | 0.029215 |
| case | 5 | -0.10373 | 0.099589 | 0.18363 | 0.14831 | 0.017232 |
| case | 6 | 0.18234 | -0.01827 | 0.02075 | 0.013675 | 0.004448 |
| case | 7 | 0.162296 | -0.00915 | -0.08039 | -0.01061 | -0.05989 |
| case | 8 | 0.153302 | -0.02282 | -0.03465 | 0.00428 | -0.03525 |
| case | 9 | -0.16061 | 0.331513 | -0.2237 | -0.12747 | 0.101379 |
| case | 10 | -0.10944 | 0.235538 | -0.16709 | -0.0784 | 0.02965 |
| case | 11 | 0.146386 | -0.06073 | 0.005681 | 0.004386 | 0.012765 |
| case | 12 | -0.11142 | 0.19983 | -0.16744 | 0.004129 | 0.01208 |
| case | 13 | 0.185967 | -0.03366 | -0.0409 | 1.37E-05 | -0.01528 |
| case | 14 | -0.12367 | 0.352758 | -0.18345 | -0.03519 | 0.121792 |
| case | 15 | 0.176185 | -0.04268 | 0.047887 | -0.04279 | 0.01905 |
| case | 16 | 0.167917 | -0.05218 | 0.073995 | -0.00787 | -0.0506 |
| case | 17 | 0.260401 | -0.0548 | -0.04477 | -0.05197 | 0.02775 |
| case | 18 | 0.136236 | -0.01198 | -0.08143 | 0.01973 | -0.03842 |
| case | 19 | 0.138834 | -0.04032 | -0.08193 | 0.042176 | -0.04385 |
| case | 20 | 0.16192 | -0.01953 | 0.072845 | -0.01953 | 0.011575 |
| control | 21 | -0.07831 | -0.00629 | 0.33192 | -0.25534 | -0.04862 |
| control | 22 | -0.07616 | 0.249194 | -0.22979 | -0.02375 | -0.01943 |
| control | 23 | -0.13902 | 0.197788 | 0.502626 | -0.41216 | -0.22149 |
| control | 24 | -0.09599 | 0.14991 | 0.305348 | -0.19437 | -0.03666 |
| control | 25 | 0.143961 | -0.00363 | -0.104 | 0.027118 | -0.02306 |
| control | 26 | -0.09239 | 0.01684 | 0.102311 | 0.238025 | -0.28501 |
| control | 27 | -0.15317 | 0.028743 | 0.200795 | 0.517263 | -0.1524 |
| control | 28 | 0.18966 | -0.06487 | 0.01809 | -0.04872 | 0.015662 |
| control | 29 | 0.15528 | 0.004804 | 0.012977 | 0.00953 | 0.03922 |
| control | 30 | -0.23502 | -0.26695 | -0.0693 | -0.16343 | 0.074389 |
| control | 31 | -0.19001 | -0.11528 | -0.13345 | -0.04037 | 0.058417 |
| control | 32 | -0.12656 | -0.00438 | 0.108747 | 0.426766 | -0.25183 |
| control | 33 | -0.18962 | -0.22214 | -0.08606 | -0.01392 | 0.072195 |
| control | 34 | -0.1854 | -0.25277 | -0.0294 | -0.05366 | 0.001843 |
| control | 35 | -0.23123 | -0.36901 | -0.06085 | -0.12174 | 0.056355 |
| control | 36 | 0.166858 | -0.00731 | 0.008392 | 0.012516 | 0.022454 |
| control | 37 | -0.18617 | -0.28143 | -0.05671 | -0.08427 | 0.021525 |
| control | 38 | -0.16842 | -0.19215 | -0.14976 | 0.001365 | -0.02457 |
| control | 39 | 0.131189 | 0.046621 | -0.12077 | -0.01231 | 0.02645 |
| control | 40 | -0.08314 | 0.055542 | 0.296105 | 0.30465 | 0.771395 |
