# Supplementary material for: Three Novel Players: PTK2B, SYK, and TNFRSF21 Were Identified to Be Involved in the Regulation of Bovine Mastitis Susceptibility via GWAS and Post-transcriptional Analysis
Source: Front Immunol. 2019 Aug 6;10:1579. doi: 10.3389/fimmu.2019.01579 (PMC6691815; doi:10.3389/fimmu.2019.01579)
Supplement: Table S3 — Three genotypic risk estimation data of case-control by Armitage-test. [file Table_3.DOCX]

|  | case | control | case + control | case/(case + control) |
| --- | --- | --- | --- | --- |
| 0 | 26013 | 25686 | 51699 | 0.503163 |
| 1 | 33707 | 31986 | 65693 | 0.513099 |
| 2 | 124414 | 122700 | 247114 | 0.503468 |
