# Supplementary material for: Three Novel Players: PTK2B, SYK, and TNFRSF21 Were Identified to Be Involved in the Regulation of Bovine Mastitis Susceptibility via GWAS and Post-transcriptional Analysis
Source: Front Immunol. 2019 Aug 6;10:1579. doi: 10.3389/fimmu.2019.01579 (PMC6691815; doi:10.3389/fimmu.2019.01579)
Supplement: Table S4 — Forty two important SNPs screened out via Bayesian Analysis Model. [file Table_4.DOCX]

| Ref_ID | Pvalue | CHISQ | OR | L95 | U95 |
| --- | --- | --- | --- | --- | --- |
| AC_000159.1-114843903 | 0.000628 | 11.69 | 0.1678 | 0.05817 | 0.4842 |
| AC_000159.1-30756917 | 0.0006931 | 11.51 | 18 | 2.182 | 148.5 |
| AC_000159.1-38937721 | 0.0009363 | 10.95 | 0.05785 | 0.006951 | 0.4815 |
| AC_000159.1-47045687 | 0.0001064 | 15.02 | 14.21 | 2.937 | 68.76 |
| AC_000159.1-98302192 | 0.0004058 | 12.51 | 0 | 0 | nan |
| AC_000160.1-90835937 | 0.0006704 | 11.57 | 5.808 | 2.038 | 16.55 |
| AC_000162.1-37588412 | 0.0008424 | 11.15 | 6 | 1.994 | 18.06 |
| AC_000162.1-5881560 | 0.0008765 | 11.07 | 0.1406 | 0.04076 | 0.4852 |
| AC_000162.1-8678060 | 0.0004653 | 12.25 | 6.929 | 2.196 | 21.86 |
| AC_000165.1-75762330 | 0.000259 | 9.078 | 7.07 | 1.78 | 28.08 |
| AC_000165.1-88640083 | 0.000192 | 5.477 | 9.25 | 1.047 | 81.7 |
| AC_000166.1-17176625 | 0.0005117 | 12.07 | 6.662 | 2.185 | 20.31 |
| AC_000166.1-17514753 | 0.0001261 | 14.7 | 0.1146 | 0.03528 | 0.3725 |
| AC_000166.1-17518215 | 0.0005613 | 11.9 | 0.1605 | 0.05443 | 0.4733 |
| AC_000166.1-22015303 | 0.0002466 | 13.44 | 0.1235 | 0.03669 | 0.4155 |
| AC_000166.1-98519900 | 0.0005384 | 11.98 | 6.25 | 2.131 | 18.33 |
| AC_000169.1-14802054 | 0.0006837 | 11.53 | 6 | 2.058 | 17.5 |
| AC_000170.1-25949166 | 2.68E-05 | 17.64 | 12.33 | 3.262 | 46.63 |
| AC_000171.1-33866959 | 0.0003664 | 12.7 | 7.222 | 2.309 | 22.59 |
| AC_000172.1-49124945 | 0.0009021 | 11.02 | 0 | 0 | nan |
| AC_000173.1-48577224 | 0.0003884 | 12.59 | 6 | 2.154 | 16.71 |
| AC_000173.1-49099498 | 7.08E-05 | 15.79 | 14.57 | 3.059 | 69.4 |
| AC_000173.1-57070376 | 0.000424 | 12.42 | 0 | 0 | nan |
| AC_000173.1-77887746 | 0.0006317 | 11.68 | 0.163 | 0.05541 | 0.4793 |
| AC_000175.1-21068792 | 0.0005112 | 12.07 | NA | NA | NA |
| AC_000175.1-27017918 | 0.0002646 | 13.31 | 20.28 | 2.496 | 164.8 |
| AC_000175.1-32265465 | 0.0003413 | 12.83 | 8 | 2.344 | 27.31 |
| AC_000175.1-41064844 | 0.0003088 | 13.02 | 20.22 | 2.488 | 164.3 |
| AC_000177.1-9704351 | 0.0006279 | 11.69 | 0.09167 | 0.01908 | 0.4404 |
| AC_000178.1-13685463 | 0.001057 | 10.73 | 0 | 0 | nan |
| AC_000178.1-27526056 | 0.0007534 | 11.35 | 17.73 | 2.126 | 147.8 |
| AC_000180.1-20438858 | 0.0006203 | 11.71 | 0.1515 | 0.04821 | 0.4762 |
| AC_000180.1-22490040 | 0.0001853 | 13.97 | 8.061 | 2.545 | 25.53 |
| AC_000180.1-26414259 | 0.0005384 | 11.98 | 0.1261 | 0.03587 | 0.443 |
| AC_000180.1-28580132 | 0.0005616 | 11.9 | 7.143 | 2.199 | 23.21 |
| AC_000181.1-3233588 | 0.0006203 | 11.71 | 6.6 | 2.1 | 20.74 |
| AC_000181.1-57506421 | 0.0006975 | 11.5 | 0 | 0 | nan |
| AC_000181.1-58979699 | 0.0006975 | 11.5 | 0 | 0 | nan |
| AC_000182.1-1988979 | 8.24E-05 | 15.5 | 11.62 | 2.975 | 45.43 |
| AC_000186.1-17424612 | 0.0004447 | 12.33 | 11.45 | 2.374 | 55.26 |
| AC_000187.1-15520017 | 0.0007293 | 11.41 | 17.7 | 2.141 | 146.3 |
| AC_000187.1-50888452 | 0.000937 | 10.95 | 0.1667 | 0.05543 | 0.5011 |
