# Supplementary material for: Three Novel Players: PTK2B, SYK, and TNFRSF21 Were Identified to Be Involved in the Regulation of Bovine Mastitis Susceptibility via GWAS and Post-transcriptional Analysis
Source: Front Immunol. 2019 Aug 6;10:1579. doi: 10.3389/fimmu.2019.01579 (PMC6691815; doi:10.3389/fimmu.2019.01579)
Supplement: Table S5 — The logistic regression model screened out 51 important SNPs. [file Table_5.DOCX]

| Ref_ID | Pvalue | OR | L95 | U95 | STAT |
| --- | --- | --- | --- | --- | --- |
| AC_000158.1-149952147 | 0.008002 | 5.912 | 1.59 | 21.98 | 2.652 |
| AC_000159.1-114843903 | 0.006341 | 0.215 | 0.07128 | 0.6483 | -2.73 |
| AC_000159.1-33714844 | 0.008363 | 7.847 | 1.697 | 36.28 | 2.637 |
| AC_000159.1-47045687 | 0.004718 | 11.36 | 2.106 | 61.32 | 2.826 |
| AC_000160.1-90835937 | 0.007839 | 3.838 | 1.424 | 10.34 | 2.659 |
| AC_000160.1-92147051 | 0.005465 | 6.554 | 1.74 | 24.69 | 2.778 |
| AC_000161.1-114599027 | 0.005104 | 7.417 | 1.825 | 30.15 | 2.8 |
| AC_000161.1-44999544 | 0.00976 | 0.1526 | 0.03666 | 0.635 | -2.584 |
| AC_000161.1-49037069 | 0.004295 | 0.07376 | 0.01233 | 0.4415 | -2.856 |
| AC_000162.1-37588412 | 0.009236 | 4.128 | 1.42 | 12 | 2.603 |
| AC_000162.1-5881560 | 0.005781 | 0.1412 | 0.03515 | 0.5669 | -2.76 |
| AC_000162.1-8678060 | 0.00522 | 5.866 | 1.695 | 20.3 | 2.793 |
| AC_000163.1-105010360 | 0.009633 | 5.424 | 1.508 | 19.51 | 2.589 |
| AC_000165.1-1297965 | 0.008168 | 0.1438 | 0.03415 | 0.6051 | -2.645 |
| AC_000165.1-14077448 | 0.002635 | 0.07926 | 0.01519 | 0.4136 | -3.007 |
| AC_000165.1-75762330 | 0.00127 | 6.605 | 1.496 | 29.15 | 2.492 |
| AC_000165.1-88640083 | 0.00314 | 12 | 1.248 | 115.4 | 2.152 |
| AC_000166.1-17176625 | 0.0069 | 4.749 | 1.534 | 14.71 | 2.702 |
| AC_000166.1-17514753 | 0.00321 | 0.03618 | 0.003978 | 0.329 | -2.947 |
| AC_000166.1-17518215 | 0.005858 | 0.05012 | 0.005962 | 0.4213 | -2.756 |
| AC_000166.1-22015303 | 0.002015 | 0.1079 | 0.02628 | 0.4434 | -3.088 |
| AC_000166.1-24287014 | 0.00998 | 0.16 | 0.03968 | 0.645 | -2.577 |
| AC_000166.1-27319191 | 0.008545 | 10 | 1.798 | 55.63 | 2.63 |
| AC_000166.1-98519900 | 0.007809 | 3.866 | 1.428 | 10.47 | 2.66 |
| AC_000167.1-13159595 | 0.007057 | 0.1612 | 0.04275 | 0.6082 | -2.694 |
| AC_000169.1-14802054 | 0.005923 | 8.018 | 1.821 | 35.32 | 2.752 |
| AC_000169.1-27514035 | 0.008269 | 0.2621 | 0.09704 | 0.7081 | -2.641 |
| AC_000169.1-63225636 | 0.005045 | 0.09471 | 0.01824 | 0.4919 | -2.804 |
| AC_000170.1-25949166 | 0.001027 | 12.78 | 2.792 | 58.52 | 3.283 |
| AC_000171.1-33866959 | 0.00748 | 4.233 | 1.47 | 12.19 | 2.675 |
| AC_000173.1-48577224 | 0.004518 | 4.881 | 1.634 | 14.58 | 2.84 |
| AC_000173.1-49099498 | 0.00866 | 7.677 | 1.676 | 35.16 | 2.625 |
| AC_000173.1-68336909 | 0.006712 | 0.08333 | 0.01382 | 0.5024 | -2.711 |
| AC_000175.1-27017918 | 0.00583 | 21.89 | 2.441 | 196.4 | 2.757 |
| AC_000175.1-32265465 | 0.006382 | 5.728 | 1.634 | 20.08 | 2.727 |
| AC_000175.1-43032228 | 0.006472 | 0.1678 | 0.04641 | 0.6064 | -2.723 |
| AC_000177.1-9704351 | 0.001736 | 0.05793 | 0.009745 | 0.3444 | -3.132 |
| AC_000180.1-11188398 | 0.009955 | 10.35 | 1.75 | 61.19 | 2.577 |
| AC_000180.1-20438858 | 0.009494 | 0.2394 | 0.08128 | 0.7052 | -2.594 |
| AC_000180.1-22490040 | 0.00419 | 6.859 | 1.836 | 25.62 | 2.863 |
| AC_000180.1-28580132 | 0.008612 | 4.642 | 1.477 | 14.59 | 2.627 |
| AC_000181.1-3233588 | 0.001532 | 12.28 | 2.603 | 57.92 | 3.169 |
| AC_000182.1-1988979 | 0.006471 | 6.124 | 1.662 | 22.57 | 2.723 |
| AC_000182.1-2100464 | 0.008973 | 4.17 | 1.429 | 12.17 | 2.613 |
| AC_000183.1-7664844 | 0.003597 | 13.36 | 2.333 | 76.47 | 2.912 |
| AC_000184.1-19690660 | 0.009672 | 0.1255 | 0.02604 | 0.6045 | -2.587 |
| AC_000184.1-35471138 | 0.00709 | 0.1663 | 0.04506 | 0.6138 | -2.693 |
| AC_000186.1-17892413 | 0.009052 | 5.587 | 1.535 | 20.34 | 2.61 |
| AC_000187.1-114647668 | 0.006258 | 0.1288 | 0.02965 | 0.5598 | -2.734 |
| AC_000187.1-13639838 | 0.004851 | 0.04062 | 0.004371 | 0.3774 | -2.817 |
| AC_000187.1-50888452 | 0.008504 | 0.22 | 0.07121 | 0.6795 | -2.631 |
