# Supplementary material for: Three Novel Players: PTK2B, SYK, and TNFRSF21 Were Identified to Be Involved in the Regulation of Bovine Mastitis Susceptibility via GWAS and Post-transcriptional Analysis
Source: Front Immunol. 2019 Aug 6;10:1579. doi: 10.3389/fimmu.2019.01579 (PMC6691815; doi:10.3389/fimmu.2019.01579)
Supplement: Table S6 — Five SNPs associated with three important SNPs screened by the Bayesian Model and Logical regression analysis model. [file Table_6.DOCX]

| Ref_ID | Chromosome | Bayesian Analysis | | | | |  | Logical Regression Analysis | | | | |
| --- | --- | --- | --- | --- | --- | --- | --- | --- | --- | --- | --- | --- |
|  |  | P* | CHISQ** | OR | L95 | U95 |  | P* | OR | L95 | U95 | STAT*** |
| rs77816736 | AC_000165.1 | 0.15 | 2.072 | 0.5065 | 0.1997 | 1.285 |  | 0.0888 | 0.3158 | 0.08374 | 1.191 | -1.702 |
| rs85635916 | AC_000165.1 | 0.01145 | 6.394 | NA | NA | NA |  | 0.9984 | 2.79E+09 | 0 | inf | 0.001995 |
| rs85927029 | AC_000165.1 | 0.1279 | 2.318 | 2.193 | 0.7914 | 6.077 |  | 0.1555 | 2.087 | 0.756 | 5.764 | 1.42 |
| rs16711445 | AC_000180.1 | 0.592 | 0.2872 | 0.6042 | 0.0942 | 3.875 |  | 0.5789 | 0.5778 | 0.08326 | 4.009 | -0.555 |
| rs19736020 | AC_000180.1 | 0.8726 | 0.02571 | 1.258 | 0.07562 | 20.93 |  | 0.871 | 1.267 | 0.07303 | 21.97 | 0.1624 |
