# Supplementary material for: Three Novel Players: PTK2B, SYK, and TNFRSF21 Were Identified to Be Involved in the Regulation of Bovine Mastitis Susceptibility via GWAS and Post-transcriptional Analysis
Source: Front Immunol. 2019 Aug 6;10:1579. doi: 10.3389/fimmu.2019.01579 (PMC6691815; doi:10.3389/fimmu.2019.01579)
Supplement: Table S7 — Three pairs of PCR amplification primers for population genetics validation. [file Table_7.DOCX]

| Primer name | Sequence (5’-3’) | Base number (bp) | Annealing temperature (℃) |
| --- | --- | --- | --- |
| SYK-F | GCTCTGCATGTGCCTCTCCT | 235 | 58 |
| SYK-R | GCCCTCTGTGCTCCTCTGTC |  |  |
| PTK2B-F | GCTCCACCATTCCTTAGTC | 430 | 58 |
| PTK2B-R | CTGGCTCCAACTATACCAC |  |  |
| TNFRSF21-F | CTGAGTAGTATTCCACCGT | 370 | 58 |
| TNFRSF21-R | ATGATCCCCACTAACTCCC |  |  |
