# Supplementary material for: Three Novel Players: PTK2B, SYK, and TNFRSF21 Were Identified to Be Involved in the Regulation of Bovine Mastitis Susceptibility via GWAS and Post-transcriptional Analysis
Source: Front Immunol. 2019 Aug 6;10:1579. doi: 10.3389/fimmu.2019.01579 (PMC6691815; doi:10.3389/fimmu.2019.01579)
Supplement: Table S8 — Primers of internal reference gene (β-actin), three immune-regulate genes, TLR4, AKT1, NF-κB, and three interleukin genes for RT-qPCR analysis. [file Table_8.DOCX]

| Primer name | Sequence (5’-3’) | Base number (bp) | Annealing temperature (℃) |
| --- | --- | --- | --- |
| mβ-Actin-F | CCTCACGGAACGTGGTTACA | 87 | 57 |
| mβ-Actin-R | TCCTTGATGTCACGCACAATTT |  |  |
| mSYK-F | ACAAGGACAAAACGGGGAAG | 258 | 59 |
| mSYK-R | TGGCTCGTAAGGGTTGAATG |  |  |
| mPTK2B-F1 | GACCTATCGCTGTGAACTC | 282 | 59 |
| mPTK2B-R1 | GACCTTGTGCTCTCCTTG |  |  |
| mTNFRSF21-F | GAAGGCACCAAGTCTCATT | 186 | 58 |
| mTNFRSF21-R | GCATTCTCGGTCAGTCAA |  |  |
| mIL8-F | CTGCCTAAACCCCAAGGAA | 206 | 59 |
| mIL8-R | AACCCTACACCAGACCCACA |  |  |
| mTLR4-F | CAGGGCAGGGAAAGTCAA | 203 | 58 |
| mTLR4-R | AGGAAAAGTGAGCCAAGACC |  |  |
| mIL1β-F | CAACCGTACCTGAACCCAT | 231 | 59 |
| mIL1β-R | GCCACGATGACCGACACCA |  |  |
| mIL-10-F | GTATCCACTTGCCAACCA | 237 | 59 |
| mIL-10-R | CGTGCTCCTTGATGTCAG |  |  |
| mAKT1-F | CCTCCTGAAGAACGATGG | 148 | 57 |
| mAKT1-R | GCGGATGATGAAGGTGTT |  |  |
| mNF-κB-F | CAGTATGCCATTGTGTTCC | 152 | 57 |
| mNF-κB-R | TCCTCCTTGTCTTCTACCA |  |  |
