# Supplementary material for: Three Novel Players: PTK2B, SYK, and TNFRSF21 Were Identified to Be Involved in the Regulation of Bovine Mastitis Susceptibility via GWAS and Post-transcriptional Analysis
Source: Front Immunol. 2019 Aug 6;10:1579. doi: 10.3389/fimmu.2019.01579 (PMC6691815; doi:10.3389/fimmu.2019.01579)
Supplement: Table S9 — Fst values of 27 significantly different SNPs. [file Table_9.DOCX]

| Ref_ID | Chromosome | Fst value |
| --- | --- | --- |
| rs47045687 | AC_000159.1 | 0.3242 |
| rs114843903 | AC_000159.1 | 0.2708 |
| rs90835937 | AC_000160.1 | -0.025 |
| rs5881560 | AC_000162.1 | 0.2512 |
| rs8678060 | AC_000162.1 | 0.2679 |
| rs37588412 | AC_000162.1 | -0.0163 |
| rs75762330 | AC_000165.1 | 0.2165 |
| rs88640083 | AC_000165.1 | 0.1297 |
| rs17176625 | AC_000166.1 | 0.1346 |
| rs17514753 | AC_000166.1 | 0.3503 |
| rs17518215 | AC_000166.1 | 0.2831 |
| rs22015301 | AC_000166.1 | 0.2763 |
| rs98519900 | AC_000166.1 | 0.2791 |
| rs14802054 | AC_000169.1 | -0.0092 |
| rs25949166 | AC_000170.1 | 0.3447 |
| rs33866959 | AC_000171.1 | -0.0034 |
| rs48577224 | AC_000173.1 | 0.2745 |
| rs49099498 | AC_000173.1 | 0.3189 |
| rs27017918 | AC_000175.1 | 0.0412 |
| rs32265465 | AC_000175.1 | 0.2778 |
| rs9704351 | AC_000177.1 | 0.2716 |
| rs20438858 | AC_000180.1 | 0.2459 |
| rs22490040 | AC_000180.1 | 0.3363 |
| rs28580132 | AC_000180.1 | 0.2852 |
| rs3233588 | AC_000181.1 | 0.2584 |
| rs1988979 | AC_000182.1 | 0.3443 |
| rs50888452 | AC_000187.1 | 0.2676 |
