# Supplementary material for: Three Novel Players: PTK2B, SYK, and TNFRSF21 Were Identified to Be Involved in the Regulation of Bovine Mastitis Susceptibility via GWAS and Post-transcriptional Analysis
Source: Front Immunol. 2019 Aug 6;10:1579. doi: 10.3389/fimmu.2019.01579 (PMC6691815; doi:10.3389/fimmu.2019.01579)
Supplement: Data Sheet 1 — A supplement to the “Materials and Methods” section. [file Data_Sheet_1.pdf]

Raw Reads quality control

The original sequencing (Raw Data or Raw Reads) gained using the Illumina HiSeq sequencing platform. The Phred value was a role of sequence base error rate. It gained by calculating the probability model of prediction base recognition error base recognition. The calculation formula was: $Q_{Phred} = -10\log_{10}$ (Phred)（Table S1）. Then deleted the reads contained the junction sequence and N base ratio ≥8% reads，got Clean Reads, then spliced by Pear (Version 0.9.6) software (http://pear.php.net/package/HTTP_WebDAV_Client/download/0.9.6/). Based on locating each sample, high-quality Enzyme Reads containing cleavage recognition sites extracted. SOAP (version 2.21, Short Oligonucleotide Analysis Package) (http://soap.genomics.org.cn/soapaligner.html) software used to align Enzyme Reads with the reference sequence. Unique tags gained by the same Reads clustering showed the sequencing depth of the tags.

Supplemental statistical model

The SNPs associated statistic obeys the multivariate normal distribution. We also calculated the likelihood of possible causal states of the SNP. Each SNP has two potential causal: effect or no effect of the SNP. Therefore, for a possible subset of each, we need to consider the 2n likelihood of the SNP. For each of these states, a multivariate normal distribution used to calculate the probability of the data for a given causal state. Thus, to identify the best SNPs set, a large amount of computations must be performed.

Principal analysis method (PCA)

PCA is a method that uses for dimensionality reduction on data to study how to condense many original into a few factors with minimal information loss (Li et al., 2017). In this experiment, let F_1 denote the main sub-index formed by the first linear combination of the original, $F_1=a_{11}X_1+ a_{12}X_1+ \ldots\ldots +a_{1m}X_m$, (m stands for the m*th* index). Information obtained by each principal component can measure by its variance. The larger the variance, the more information the F contains. If the first principal component is not enough to represent the initial m indicators, then consider

selecting the second index F_2. The existing data of F_1 does not need to appear in F_2 again. That is, F_2 and F_1 should be independent, irrelevant, and expressed by their covariance. And so on to build F_1, F_2… F_n, as equation (1).

$$\begin{cases} F_1 = a_{11}X_1 + a_{12}X_2 + \dots\dots + a_{1m}X_m \\ F_2 = a_{21}X_1 + a_{22}X_2 + \dots\dots + a_{2m}X_m \\ \dots\dots \\ F_n = a_{n1}X_1 + a_{n2}X_2 + \dots\dots + a_{nm}X_m \end{cases} \quad (1)$$

Bayesian and Logistic regression model association Analysis

Linear models are a common method for correlation analysis of phenotypes and genotypes. Strict quality control used to remove poorly performing SNP marker loci in RAD typing. Bayesian and Logistic regression model introduced for GWAS detected SNPs associated with clinical mastitis in dairy cows. First, built the following linear regression equation based on phenotype (Guo et al., 2018):

$$y_i = \mu + \sum_{k=1}^{M} X_{ik}\alpha_k + e \quad (2)$$

Where y_i is a vector of phenotype for individual i; M is the number of SNPs; μ is a vector of the overall mean of traits phenotypes; α_k is a vector of additive correlation effect of the kth SNPs; Xik is a vector of the genotype (0, 1, or 2) of the kth SNPs observed on the ith individual; and e is a vector of residual effect.

The Bayesian model assumed the SNPs effect was a prior normal distribution. Firstly, we consider the possibility that each SNP locus truly associated with the mastitis phenotype in GWAS. Select a value п for the prior probability H_1. The correlation between SNPs and dairy mastitis traits quantified using п values. A pre-estimation of the SNPs truly associated with cow mastitis trait performed by a specific п value (10^{-4}-10^{-6}). While the probability of H_0 considered to be (1-п). Secondly, calculated the Bayes factor for each SNPs. The Bayesian factor (BF) is the ratio between the probability of data at H_1 and H_0. Null assumption is H_0 ($\theta_{het} = \theta_{hom} = 0$). H_1, at least one $\theta_{het}=t_1$ and $\theta_{hom}=t_2$ value is non-zero (Wellcome Trust Case Control et al., 2012).

$$BF = \frac{P(data \mid \theta_{het}=t_1, \theta_{hom}=t_2)}{P(data \mid \theta_{het}=0, \theta_{hom}=0)} \quad (3)$$

Where, θ_{het} is odds ratios (ORs) logarithm between the heterozygote and the common homozygote. θ_{hom} is the ORs logarithm between rare and common

homozygotes. Then counted the posterior odds (PO) under the H_1 condition: PO= BF×п/ (1-п). And posterior probability of association (PPA= PO/ (1+PO)) can be regarded as a Bayesian simulation of P value.

The SNPs effects variances were independent of each other, and each of which followed the same independent distribution (IID) as the inverse chi-square prior normal distribution where v is a parameter of the degree of freedom and S^2 the parameter of scale:

$$P(\sigma_k^2) = \chi^{-2}(\sigma_k^2 \mid v, S^2) \qquad (4)$$

A prior distribution of the criticality of each SNP effect was a t-distribution (Meuwissen et al., 2001;Guo et al., 2018):

$$P(\alpha_k \mid v, S^2) = \int N(\alpha_k \mid 0, \sigma_k^2)\, \chi^{-2}(\sigma_k^2 \mid v, S^2) d\sigma_k^2 \qquad (5)$$

The prior for a_k depends on the variance of each SNPs, and each variance has an inverse Chi-square. SNP has null effect with probability п or is a normal distribution with probability (1-п), N $(0,\sigma_a^2)$ (Gianola, 2013):

$$a_k \mid \pi,\ \sigma_a^2 = \begin{cases} N(0, \sigma_a^2)\ with\ probability\ (1-\pi) \\ 0 \quad with\ probability \quad \pi \end{cases} \qquad (6)$$

Where σ_a^2 is represents the common variance of all non-zero SNPs effects, and it prorated prior distribution of the chis-square, $\chi^{-2}(v_a, S_a^2)$. The unknown п value in the model predicted from its prior distribution (considered as uniform between 0 and 1) or п– uniform (0, 1).

v_a is designated as 4, S_a^2 is calculated by additive variance.

$$S_a^2 = \frac{\tilde{\sigma}_a^2(v_a-2)}{v_a} \qquad (7)$$

$$\text{and } \tilde{\sigma}_a^2 = \frac{\tilde{\sigma}_S^2}{(1-\pi)\sum_{k=1}^{K} 2P_k(1-P_k)} \qquad (8)$$

Where the allele frequency of the kth SNP isP_k, the variance of a given tag is$\tilde{\sigma}_a^2$, and the additive genetic variance $\tilde{\sigma}_S^2$ is elucidated by SNPs.

Then assuming that SNPs affects the mastitis phenotypic traits, we constructed logistic regression equation to predict SNPs associated with clinical mastitis in dairy cows. And we established a fitted logistic regression equation (Biscarini et al., 2016;Wang et al., 2016):

$$\log(Pj/(1-Pj)) = \mu + \sum_{j=1}^{M} X_{ij}\beta_j \qquad (9)$$

Where, P_j is the probability of occurrence of clinical phenotype under a condition X_{ij}; $(1\text{-}p_j)$ is the probability that phenotype does not occur; $X_{ij} = (X_{1j}, X_{2j}, X_{3j} \ldots\ldots X_{mj})$ is the genotype of individual i at position j (0, 1, or 2); β_j is the effect of jth SNPs; and m is the number of samples; μ is the overall mean of traits phenotypes.

In the logistic regression model, Y= ($\mu+\Sigma\beta_i X_i$) or ($\log P/(1-P)$), the equation can transform into another equation form:

$$P = \frac{\exp(Y)}{1+\exp(Y)} \qquad \text{or}$$

$$Log\,(P) = \beta_0+\beta_1 X_1+\ldots\ldots+\beta_i X_i \qquad (10)$$

Where P is clinical mastitis phenotype, X_i is the genotype of individual i, β_i is the odds ratio (OR). The equation of expression between P and variable X_i can derive by equation transformation:

$$P = \frac{\exp(\mu+\Sigma\beta iXi)}{1+\exp(\mu+\Sigma\beta iXi)} \qquad (11)$$

$$1-P = \frac{1}{\exp(\mu+\Sigma\beta iXi)} \qquad (12)$$

$$OR = \exp(\beta_i) = \frac{p1/(1-p1)}{p0/(1-p0)} \qquad (13)$$

The greater the value of β_i, the greater the influence of Y. 95% confidence interval: CI= exp (β_i±1.96SE（β_i）).

Case-control population verification analysis

We determined the number of validation samples using a matching design and case-control unequal (case/control=1/h).

$$n = \frac{(z_\alpha\sqrt{(1+1/h)\bar{p}(1-\bar{p})}+z_\beta\sqrt{p_1(1-p_1)/h+p_0(1-p_0)})^2}{(p_1-p_0)^2} \qquad (14)$$

$$p_1 = \frac{p_0 OR}{1+p_0(OR-1)} \qquad (15)$$

$$\bar{p} = \frac{(p_1+hp_0)}{(1+h)} \qquad (16)$$

$$OR = ad/bc \qquad (17)$$

$$OR\ 95\%\ CI = OR^{(1\pm1.96/\sqrt{\chi^2})} \qquad (18)$$

$$\chi^2 = \frac{(ad-bc)^2 N}{(a+b)(c+d)(a+c)(b+d)} \qquad (19)$$

Where, n is the number of cows in clinical mastitis. *N* is the total number of cows in verification population. P_0 is the exposure rate of SNPs in the control group. P_1 is the exposure rate of SNPs in the case group. OR is the exposure ratio (Odds ratio). α is the probability of hypothesis testing type I errors. β is the probability of hypothesis type II errors and (1- β) is the expected test assurance. OR 95% CI is 95% confidence interval.

The Attributable Fraction reflects the probability that a case will be randomly selected from the population due to the SNPs.

$$AF_e = \frac{I_e - I_0}{I_e} \approx \frac{OR-1}{OR} \qquad (20)$$

Where, I_e is the incidence of the site mutation group; I_0 is incidence of the site non-mutation group. Incidence is generally not available in case-control studies and only OR obtained. AF_e refers to the proportion of mastitis caused by the SNPs to all mastitis.

$$AF_p = \frac{I_p - I_0}{I_p} \approx \frac{p_e(OR-1)}{1+p_e(OR-1)} \qquad (21)$$

Where, AF_p indicates the proportion of mastitis caused by the SNPs in all mastitis. I_p is the total incidence of mastitis in Chinese Holstein cows. I_0 is the incidence of mastitis with non-mutation at the SNP locus. P_e is the mutation rate of SNP locus in control group.

Differential expression of genes relative quantitative Analysis based on RT-qPCR results via Student's t-test:

$$2^{-\triangle\triangle C_t} = 2^{-[(C_tE-C_tF)-(C_tA-C_tB)]} \qquad (22)$$

Where, C_tE is the mean of C_t value of the target gene in case group. C_tA is the mean C_t value of the target gene in control group. C_tF is the mean C_t value of the reference gene in case group. C_tB is the mean C_t value of the reference gene in control group.

Biscarini, F., Schwarzenbacher, H., Pausch, H., Nicolazzi, E.L., Pirola, Y., and Biffani, S. (2016). Use of SNP genotypes to identify carriers of harmful recessive mutations in cattle populations. *BMC Genomics* 17**,** 857.

Gianola, D. (2013). Priors in whole-genome regression: the bayesian alphabet returns. *Genetics* 194**,** 573-596.

Guo, P., Zhu, B., Niu, H., Wang, Z., Liang, Y., Chen, Y., et al. (2018). Fast genomic prediction of breeding values using parallel Markov chain Monte Carlo with convergence diagnosis. *BMC*

Bioinformatics 19, 3.

Li, Z., Chen, J., Yu, H., He, L., Xu, Y., Zhang, D., et al. (2017). Genome-wide association analysis identifies 30 new susceptibility loci for schizophrenia. *Nat Genet* 49, 1576-1583.

Meuwissen, T.H., Hayes, B.J., and Goddard, M.E. (2001). Prediction of total genetic value using genome-wide dense marker maps. *Genetics* 157, 1819-1829.

Wang, S., Zhang, Y., Dai, W., Lauter, K., Kim, M., Tang, Y., et al. (2016). HEALER: homomorphic computation of ExAct Logistic rEgRession for secure rare disease variants analysis in GWAS. *Bioinformatics* 32, 211-218.

Wellcome Trust Case Control, C., Maller, J.B., Mcvean, G., Byrnes, J., Vukcevic, D., Palin, K., et al. (2012). Bayesian refinement of association signals for 14 loci in 3 common diseases. *Nat Genet* 44, 1294-1301.
